# Incorporating mRNA therapeutics into biological treatments of hematologic malignancies

**DOI:** 10.3389/fimmu.2025.1680071

**Published:** 2025-10-22

**Authors:** Jaromir Hunia, Jaromir Tomasik, Natalia Czerwik, Parmida Sadat Pezeshki, Dominika Nowis

**Affiliations:** ^1^ Laboratory of Experimental Medicine, Medical University of Warsaw, Warsaw, Poland; ^2^ Doctoral School, Medical University of Warsaw, Warsaw, Poland; ^3^ Department of Hematology, Transplantation and Internal Medicine, Medical University of Warsaw, Warsaw, Poland; ^4^ Department of Immunology, Medical University of Warsaw, Warsaw, Poland; ^5^ School of Medicine, Tehran University of Medical Sciences, Tehran, Iran; ^6^ International Hematology/Oncology of Pediatric Experts (IHOPE), Universal Scientific Education and Research Network (USERN), Tehran, Iran

**Keywords:** mRNA technology, hematologic malignancies, CAR-T cells, bispecific antibodies, monoclonal antibodies, artificial intelligence in hematology

## Abstract

The recent advancement of mRNA technology has opened new therapeutic avenues for treating hematologic malignancies, offering innovative approaches to enhance existing immunotherapies. This review examines the expanding role of *in vitro* transcribed (IVT)-mRNA-based platforms in hemato-oncology, focusing on key areas: monoclonal antibody production, bispecific antibody development, and CAR-T cell engineering. Unlike conventional biologics, mRNA allows for *in vivo* expression of therapeutic proteins, reducing manufacturing complexity and expanding access through scalable, cell-free synthesis. IVT-mRNA-encoded monoclonal and bispecific antibodies can overcome limitations such as short half-life and the need for continuous infusion, while enabling innovations like Fc silencing, protease-activated masking, and combinatorial immunotherapies. In CAR-T cell therapy, IVT-mRNA provides transient, safer alternatives to viral vector-based approaches and facilitates emerging strategies such as *in vivo* CAR programming and IVT-mRNA vaccine-like boosters. Despite these advantages, challenges remain, including delivery precision, durability of therapeutic effects, and limited clinical trial success. Beyond therapeutic mechanisms, the integration of bioinformatics and AI in IVT-mRNA design is accelerating the development of personalized and efficient cancer treatments. Overall, mRNA technology is redefining immunotherapy in hematology and holds the potential to broaden access to advanced treatments globally.

## Graphical Abstract

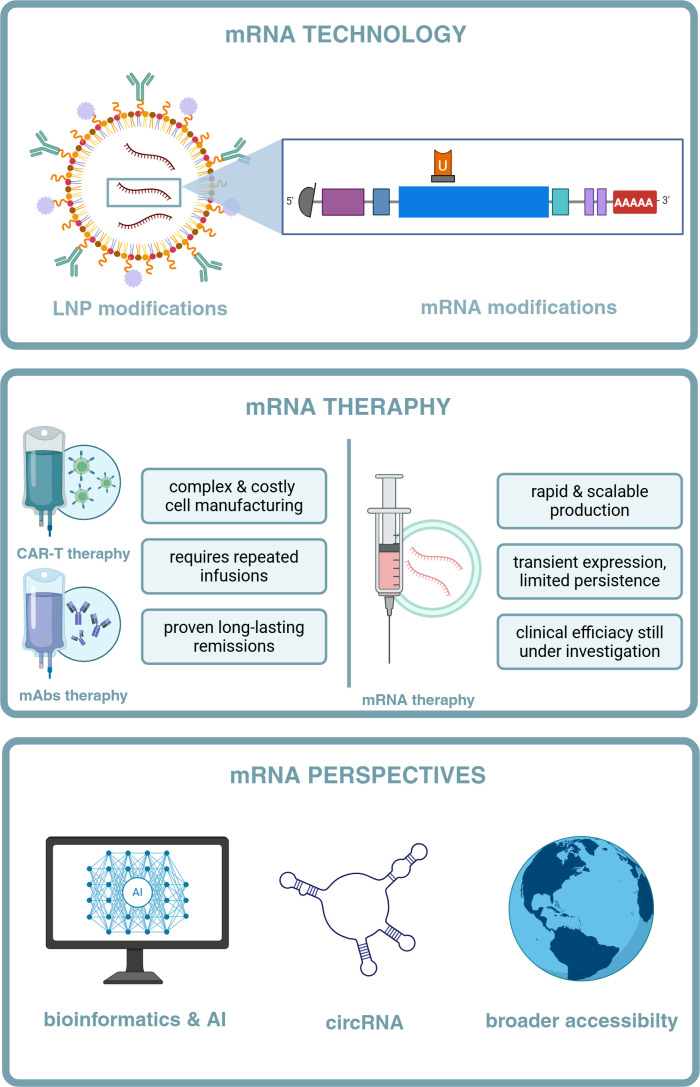

## 1 Introduction

Hematologic malignancies encompass a group of cancers that stem from lymphohematopoietic system. These malignancies include such categories as: acute and chronic leukemias, lymphomas, multiple myelomas (MM), myelodysplastic syndromes (MDS), and myeloproliferative neoplasms (MPNs). Acute lymphoblastic leukemia (ALL) is defined by an abnormal expansion of immature lymphocytes (1). The most prevalent form of acute leukemia in adults is acute myeloid leukemia (AML). It arises from hematopoietic stem cells (HSCs) or more differentiated myeloid progenitor cells, and is driven by genetic mutations that contribute to its extensive heterogeneity (2, 3). Lymphomas are a class of hematologic neoplasms that can form solid tumors. They are generally classified as either Hodgkin lymphoma (HL), which represents ca. 10% of lymphomas, or non-Hodgkin lymphoma (NHL). Among NHL subtypes, diffuse large B-cell lymphoma (DLBCL), mantle cell lymphoma (MCL), and follicular lymphoma (FL) belong to the most frequently diagnosed (4). HL displays unique histological, immunophenotypic, and clinical characteristics, with classical HL (cHL) and nodular lymphocyte predominant HL as its main forms (5). MM, MDS, and MPN are mostly diagnosed in older adults – MM alone accounts for around 10% of hematologic cancers and currently lacks a curative therapy. It often begins as silent precursors such as monoclonal gammapathy of undetermined significance (MGUS) or smoldering MM (SMM) (6). MDS, meanwhile, is a clonal disorder marked by defective hematopoiesis and an inherent risk of progression to AML (7).

Blood cancers encompass a highly diverse spectrum of diseases, posing serious risks to patients, imposing substantial burdens on healthcare systems, and presenting major challenges for the development of effective curative therapies (8).A precise understanding of these processes occurring in cancer cells is essential for designing new treatments for the diseases, that have so far remained beyond the reach of successful therapeutic outcomes.

The emergence of immunotherapy has transformed the treatment of hematologic malignancies, offering lasting remission, especially in relapsed or refractory (R/R) cases. These cancers interact constantly with immune cells, shaping an immune microenvironment that, simultaneously, supports surveillance and enables tumor survival. Originating in the immune system, they exhibit both immunostimulatory and immunosuppressive traits (9). Various immunotherapies aim to boost the body’s immune response, each with unique advantages and limitations that require further refinement.

One innovative therapeutic approach of immunotherapy is the use of mRNA technology. Following the success of *in vitro* transcribed (IVT)-mRNA-based coronavirus disease 2019 (COVID-19) vaccines, IVT-mRNA therapeutics have gained significant traction within the biopharmaceutical field. Due to their capacity for rapid production, personalization, and strong reactogenicity, IVT-mRNA applications are now being explored in oncology. Current applications of IVT-mRNA-based therapeutics in oncology can be categorized into four main areas: (1) IVT-mRNA vaccines designed to elicit immune responses against tumor-specific antigens, (2) IVT-mRNA-encoded monoclonal antibodies that enable transient *in vivo* production of antibodies, (3) IVT-mRNA-engineered chimeric antigen receptor (CAR)-T cell therapies, where IVT-mRNA is used to transiently express chimeric antigen receptors in T cells, and (4) IVT-mRNA coding for functional proteins, such as cytokines, immune checkpoint inhibitors, or pro-apoptotic factors, aimed at modulating the tumor microenvironment or directly inducing tumor cell death (10).

This review summarizes the development of IVT-mRNA therapeutics, from their early experimental foundations through the advances achieved during the COVID-19 pandemic to subsequent refinements in platform design. Applications in hematology are then considered, with attention to their integration into monoclonal antibodies (mAbs), bispecific antibodies (bsAbs), and chimeric antigen receptor (CAR) T-cell therapies. T-cell engagers (TCEs), a subclass of bsAbs, are highlighted as an example of how mRNA delivery may be applied to address current challenges. The review concludes with perspectives on future directions, including the use of artificial intelligence (AI) for molecular optimization, strategies to support scalable clinical translation, and the development of next-generation RNA formats with expanded functionality.

## 2 Principles of mRNA therapeutics

mRNA serves as a crucial intermediary in gene expression, transmitting genetic information from DNA in the nucleus to ribosomes in the cytoplasm, where proteins are synthesized. This process underlies the regulation of nearly all cellular functions (11). Beyond its natural role, mRNA is now being harnessed as a therapeutic tool, offering new strategies for treating cancer, infectious diseases, and genetic disorders.

### 2.1 Molecular design

The development of IVT was pivotal technological breakthrough for mRNA research. In 1990, Wolf et al. demonstrated that IVT of DNA into mRNA could generate transcripts capable of serving as translational templates in transfected cells. However, the resulting IVT-mRNA was inherently unstable and rapidly degraded by ubiquitous intra- and extracellular ribonucleases. The therapeutic limitation was later addressed through strategic chemical and structural modifications to the IVT-mRNA molecule, which greatly improved its stability and translational efficiency. These advances laid the foundation for the use of IVT-mRNA vaccines, gene therapies, and other innovative medical treatments (12).

The initial therapeutic aim of IVT-mRNA was to replace or supplement missing or defective proteins in patients (13). In 1992, early studies of Jirikowski et al. showed that intracerebral injecting vasopressin IVT-mRNA could partially reverse diabetes insipidus in rats (14). Soon after, IVT-mRNA was also explored as an antigen source in vaccines against infectious diseases and cancer. One of the earliest applications of IVT-mRNA in cancer immunotherapy occurred in the mid-1990s, when Gilboa’s group pioneered the use of IVT-mRNA-pulsed dendritic cells to present tumor antigens – a groundbreaking step in the development of IVT-mRNA-based cancer vaccines (15). Subsequently, it was proposed that IVT-mRNA could serve as an antigen source in vaccines for both infectious diseases and cancer, ultimately leading to the creation of IVT-mRNA vaccines (12, 16). Consequently, the European Medicines Agency (EMA) has designated IVT-mRNA-based therapeutics as Advanced Therapy Medicinal Products (ATMPs), and more specifically, as Gene Therapy Medicinal Products (GTMPs) (17).

The COVID-19 pandemic significantly boosted interest in IVT-mRNA-based therapies (18). On December 11, 2020, the U.S. Food and Drug Administration (FDA) granted emergency use authorization for the COVID-19 vaccine, Comirnaty (BNT162b2), developed by BioNTech and Pfizer using IVT-mRNA technology (19–21)., followed by the Moderna’s Spikevax (mRNA-1273), granted by FDA on December 18, 2020. Since then, IVT-mRNA vaccines have been widely administered, playing a crucial role in curbing the spread of COVID-19 globally (22). In 2022-2023, updated bivalent formulations of both Spikevax and Corminaty targeting Omicron subvariants were authorized by the FDA.^1^ (23) Beyond COVID-19, in May 2024, Moderna’s mRNA 1345 (mRESVIA) was approved by the FDA as the first IVT-mRNA-based vaccine targeting a non-COVID-19 indication, namely the prevention of respiratory syncytial virus (RSV).^2^ The platform’s versatility was further evidenced by Japan’s November 2023 approval of Arcturus/CSL’s self-amplifying Spikevax alternative, Kostaive, authorized in the European Union (EU) in February 2025.^3^

**Figure 1:**
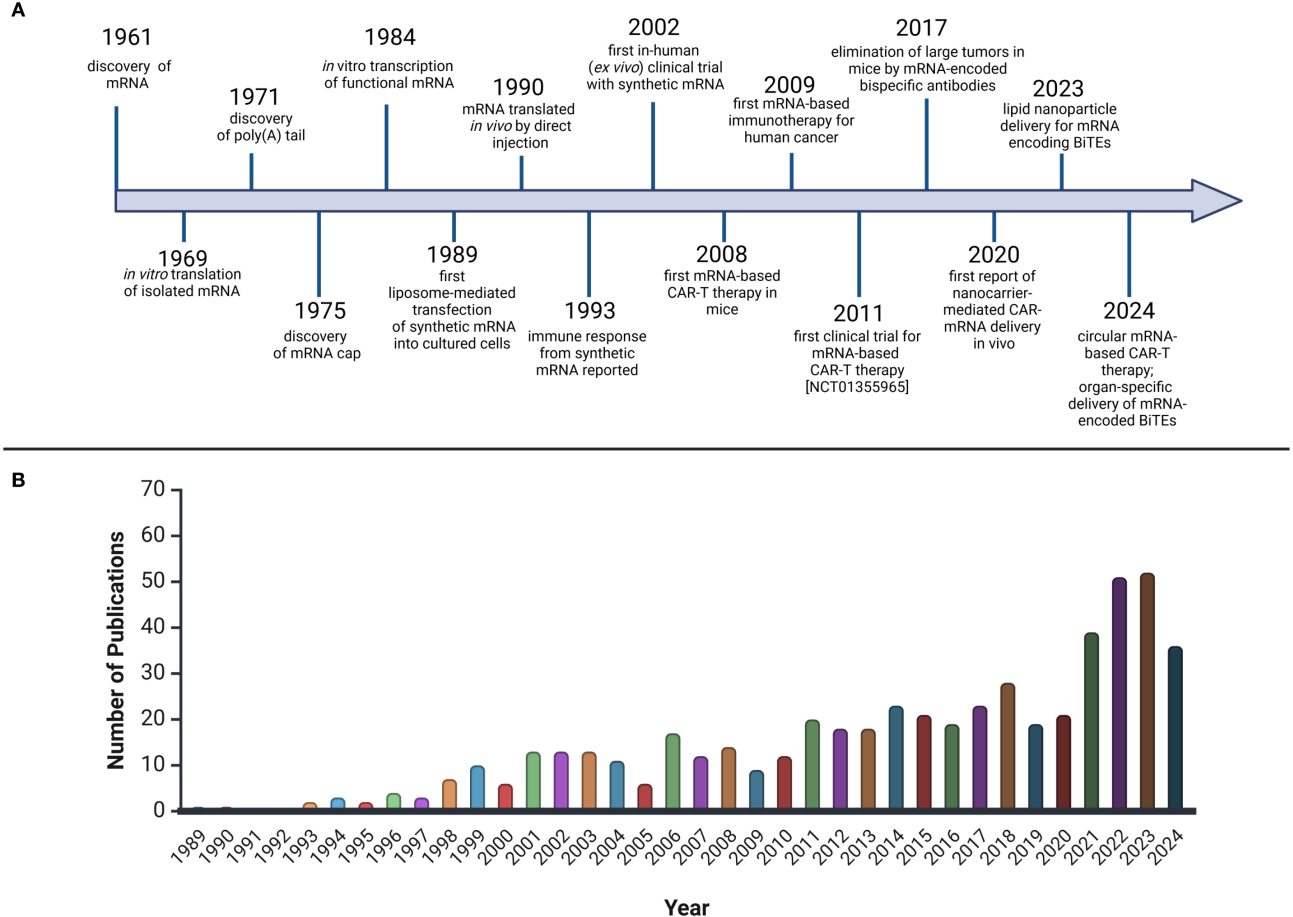
The rise and bloom of mRNA therapeutics in hematology. **(A)** Timeline of milestones in the development of mRNA for CAR-T and BiTE therapies. The development of mRNA encoding CAR-T and BiTE therapies began with the discovery of mRNA in 1961 ^58^. In 1969, the first *in vitro* translation of mRNA was achieved ^59^, followed by the discovery of the poly **(A)** tail (1971) ^60^ and the mRNA cap (1975) ^61^. In the 1980s and 1990s, techniques for mRNA transcription and transfection were developed ^62^ ^63^. In 1990, *in vivo* translation of mRNA was achieved ^64^, and by 1993, an immune response to synthetic mRNA was documented ^65^. In 2002, the first clinical trials with synthetic mRNA took place ^66^, and in 2008, CAR-T therapy was tested in mice ^67^. The first clinical CAR-T trials using mRNA began in 2011 ^68^. In 2017, tumor elimination using mRNA-encoded bispecific antibodies was achieved ^69^. In 2020, CAR-mRNA delivery was advanced with nanocarriers and lipid nanoparticles (LNPs) ^70^. By 2023, LNP-mediated delivery of mRNA encoding BiTEs was reported ^71^, and in 2024, circular mRNA-based CAR-T therapies and organ-specific delivery of mRNA-encoded BiTEs were introduced ^72^ ^73^. **(B)** Publications Mentioning mRNA Therapeutics in Hematological MalignanciesBased on PubMed, keywords: “mRNA therapeutics,” “hematological malignancies.” Created with BioRender.

The severe acute respiratory syndrome coronavirus 2 (SARS-CoV-2) pandemic highlighted the immense potential of IVT-mRNA as a therapeutic agent, driven by the urgent need for rapid vaccine development. This swift progress was made possible due to the extensive experience and advancements in mRNA technology over the past three decades (24). (**Figure 1**).

The primary sensors of the innate immune response, which play a crucial role in detecting IVT-mRNA within cells, are pattern recognition receptors (PRRs). mRNA is recognized by PRRs such as Toll-like receptors (TLRs) 3, 7, and 8, as well as retinoic acid-inducible gene I (RIG-I) and melanoma differentiation-associated protein 5 (MDA5), leading to the upregulation of pro-inflammatory cytokines and activation of the inflammasome (25, 26). TLR3 detects double-stranded RNA (dsRNA), while TLR7/8 recognizes single-stranded RNA (ssRNA) (27). Systemic administration of unmodified and unpurified IVT-mRNA can strongly activate the immune system, triggering the production of pro-inflammatory cytokines and type I interferons. This challenge arises primarily because IVT-mRNA does not follow the natural nuclear-to-cytoplasmic export pathway of endogenous mRNA, but instead enters cells *via* endocytosis and must escape from endosomes into the cytoplasm – a step that is both inefficient and a major bottleneck in IVT-mRNA delivery. Endosomes typically degrade IVT-mRNA before it can reach the cytoplasm, thus reducing its therapeutic potential (28).

To address this, several strategies have been developed to enhance endosomal escape. One approach involves the use of lipid nanoparticles (LNPs), which are engineered to protect IVT-mRNA from degradation while facilitating cellular uptake. These LNPs can be modified with ionizable lipids, which become protonated in the acidic environment of the endosome, leading to the destabilization of the endosomal membrane and enabling the mRNA to escape into the cytoplasm. This approach has proven critical in the successful delivery of IVT-mRNA vaccines and other therapeutic IVT-mRNA applications (29–31).

Another problem is that IVT-mRNA typically exhibits a different pattern of base modifications compared to the cell’s own mRNA. The pivotal discovery by Karikó and Weissman showed, that incorporation of specific nucleoside modifications allows IVT-mRNA to partially evade recognition by PRRs, thereby reducing innate immune activation while enhancing translation efficacy. For example, modifications such as pseudouridine, 2-thiouridine, 5-methylcytidine, N_1_-methylpseudouridine, or 5-methylpyridine can diminish TLR7- and TLR8-mediated sensing (32). Additionally, activation of RIG-I and protein kinase RNA-activated (PKR) can be mitigated through the introduction of pseudouridine and 2-thiouridine (33–37).

**Figure 2:**
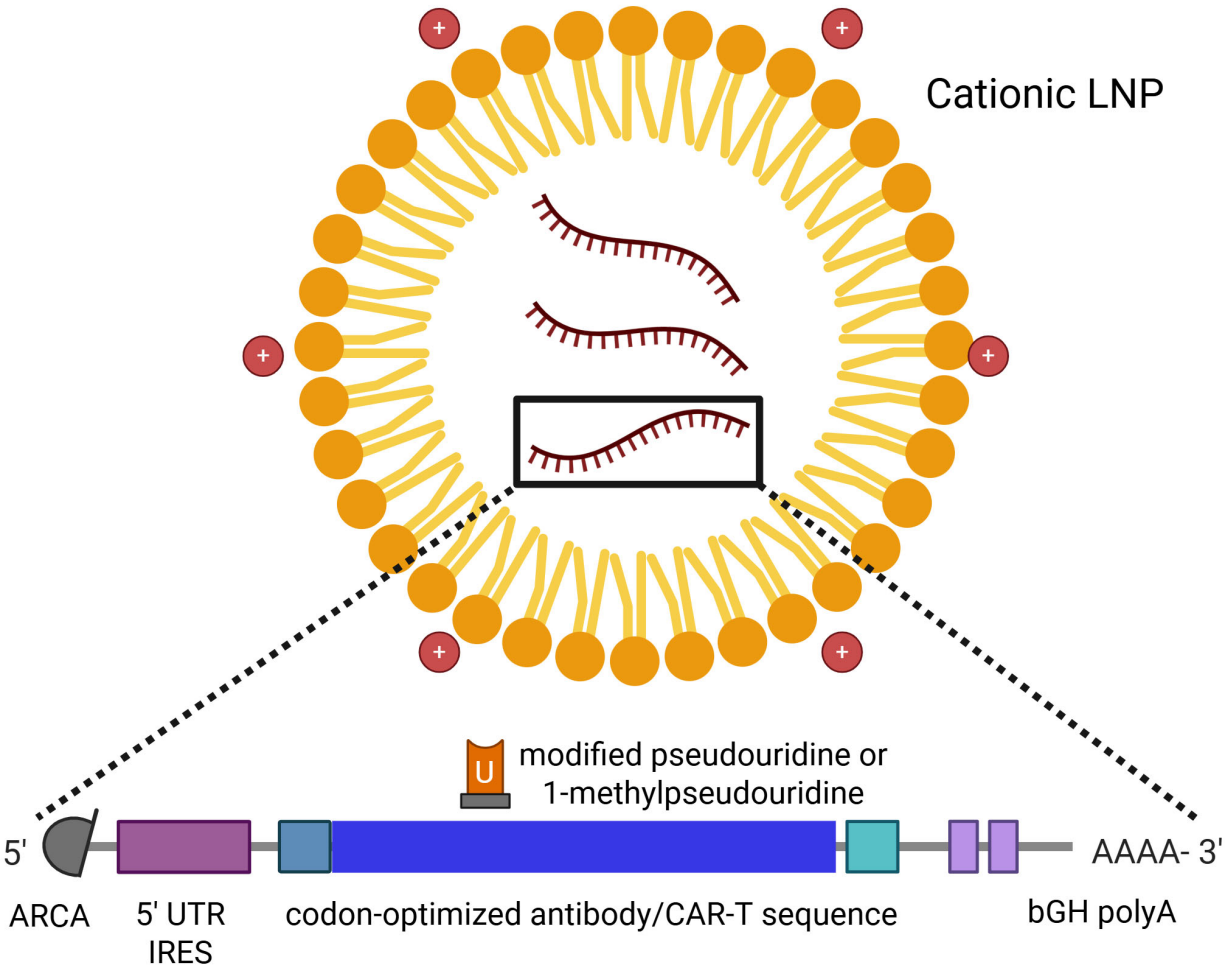
The LNP-encapsulated mRNA molecule with its modifications – 1-methylpseudouridine, 5’-UTR IRES, 5’-cap, and bGH poly-A. Created with Biorender.

Indeed, earlier studies demonstrated that replacing uridine with pseudouridine throughout the IVT-mRNA sequence could yield non-reactogenic IVT-mRNA (32, 34, 38). By combining various nucleotide substitution strategies, researchers achieved reduced activation of PRRs - such as TLR3/7/8 and RIG-I - in human peripheral blood mononuclear cells (PBMCs). The incorporation of N_1_-methylpseudouridine into IVT-mRNA molecules not only diminished their reactogenicity but also enhanced their translational efficiency both *in vitro* and *in vivo*. Chemical modification of nucleoside sites has thus emerged as a cornerstone in the optimization of therapeutic IVT-mRNA production (33, 35). However, it is important to note that while chemically modified uridines may not directly improve translational efficacy – since ribosomes may often read unmodified uridine more efficiently than its modified counterparts – the primary benefit of these modifications lies in the reduction of mRNA-induced immune activation. The decreased immune recognition prevents the activation of innate immune responses that would otherwise hinder translation and protein expression. Besides the codon-optimized coding sequence, the current literature identifies four additional key regions of IVT-mRNA that are targeted for modifications during its production (39–41): (1) the 5’ cap structure, (2) the 5’ untranslated region (UTR), (3) the 3’ UTR, and (4) the poly-A tail (**Figure 2**).

Despite these advancements, even fully modified IVT-mRNA containing optimally altered nucleosides retains some capacity to activate the immune system. Modifications do not entirely eliminate the ability of IVT-mRNA to trigger PRR sensors, partly due to impurities in the material. For instance, double-stranded RNA (dsRNA) contaminants can activate RIG-I, MDA5, PKR, and 2’-5’ oligoadenylate synthetase. High-performance liquid chromatography (HPLC) is one established method for purifying IVT-mRNA from such impurities. Purified IVT-mRNA exhibits significantly lower immunogenicity, reduced induction of type I interferons (IFNs) and tumor necrosis factor α (TNF-α), and enhanced translational capacity of the encoded proteins (16, 32, 34, 42, 43).

Even with these purification and optimization techniques, the protein products of the IVT-mRNA retain some immunogenic properties, particularly the potential to elicit anti-drug antibodies (ADA) and pro-inflammatory cytokine responses, which may interfere with the desired therapeutic outcome. However, in certain contexts, such immunogenicity can be advantageous, serving i.e. as an intrinsic adjuvant in IVT-mRNA-based vaccines (42, 44).

### 2.2 Delivery platforms

Another critical consideration is the IVT-mRNA delivery method into target cells. Like all nucleic acid-based therapeutics, IVT-mRNA faces challenges related to its negative charge, high molecular weight, and inability to passively cross the hydrophobic cell membranes. To overcome these barriers, various delivery strategies have been developed, including: (1) optimized injection protocols - e.g. intramuscular or intradermal routes that leverage local immune cells for uptake, (2) physical methods - such as electroporation or gene gun-based delivery, which facilitate cellular entry via mechanical or electrical disruption, (3) chemical complexation – with cationic polymers or protamine, which condense IVT-mRNA into more stable, positively charged particles, (4) adjuvants that enhance immunogenicity when co-delivered with IVT-mRNA, or (5) nanocarrier encapsulation, particularly LNPs, which protect IVT-mRNA from degradation and promote endosomal escape into the cytosol. LNPs, composed of four main lipid types - (1) cholesterol, (2) PEGylated lipids, (3) ionizable lipids, (4) phospholipids, and IVT-mRNA—form globular structures under acidic conditions, enabling IVT-mRNA transport to a cell in an endosome-like manner (16, 45). The first in-human study evaluating the immunogenicity and safety of LNP-encapsulated IVT-mRNA, conducted by Moderna using an influenza HA mRNA vaccine (NCT03076385), demonstrated an acceptable safety profile and sufficient immunogenicity in 2017 (46). Nevertheless, LNP formulations require further optimization, and their composition remains a focus of ongoing research aimed at developing advanced IVT-mRNA delivery systems (47–50).

IVT-mRNA-based therapeutics hold immense promise for advancing treatment strategies, particularly in infectious diseases and oncology. Infectious diseases, characterized by their rapid evolution and spread - as exemplified by the COVID-19 pandemic and other historical outbreaks - benefit from the relative ease and cost-effectiveness of IVT-mRNA production, which facilitates rapid response to emerging pathogens (16). In hemato-oncology, the diversity and individuality of cancer targets make IVT-mRNA an attractive platform for personalized therapies and precision delivery systems.

Oncology-focused IVT-mRNA therapeutics employ approaches such as genome editing, cytokine-based immunotherapy, transient ex vivo engineering of T cells, and *in vivo* production of conventional or bispecific antibodies. These strategies have the potential to reduce the toxicity associated with traditional high-dose treatments (51, 52). However, challenges remain, including delivery efficiency, durability of effects, and potential off-target immune activation.

## 3 Applications in hematology

### 3.1 Monoclonal antibodies

mAbs are pivotal components of cancer immunotherapy, functioning through multiple mechanisms to mobilize the immune system against tumor cells. These mechanisms include: 1) direct induction of programmed cell death (PCD), driving cancer cells into apoptosis, and 2) activation of immune-mediated pathways such as antibody-dependent cellular cytotoxicity (ADCC), complement-dependent cytotoxicity (CDC), and macrophage-mediated phagocytosis (53–56). These cellular pathways rely heavily on interactions between the Fc region of the antibody and the Fc gamma receptors (FcγRs) on tumor cells, making mAbs powerful therapeutic agents for targeting cancer cells in various hematologic malignancies (57, 58).

First-generation mAbs were murine-derived proteins, IgG molecules targeting single antigenic epitopes on cancer cells. These antibodies were traditionally produced using hybridoma technology, which involves fusing antigen-activated murine B lymphocytes with myeloma cells. The B lymphocyte component enables the hybridoma to secrete highly specific antibodies, while the myeloma component allows for their mass production. However, a significant drawback of these murine-derived antibodies is their potential to trigger a human anti-mouse antibody (HAMA) response, which can reduce therapeutic efficacy and increase adverse effects (59, 60). This limitation spurred the development of increasingly humanized mAbs.

Second-generation mAbs, such as chimeric antibodies (e.g., rituximab), combine murine variable regions with human constant domains, significantly reducing but not entirely eliminating the HAMA response (61, 62). Further advancements led to humanized mAbs, where only the complementarity-determining regions (CDRs) are murine, and the majority of the sequences are of human origin, further minimizing immunogenicity. Fully human mAbs exhibit the lowest immunogenicity and are produced using the following platforms: (1) phage display libraries, (2) yeast display systems, (3) transgenic mice hybridomas, (4) human hybridoma technology, (5) single B cell cloning, (6) glycoengineering. While fully human mAbs rarely induce ADAs, isolated cases of anti-idiotypic responses have been reported (63–65).

The development and implementation of mAbs in hemato-oncology have significantly expanded therapeutic options and improved clinical outcomes for many diseases. By targeting specific antigenic epitopes on cancer cells and mediating immune system activation, mAbs offer a vast array of therapeutic approaches, establishing them as a cornerstone in the fight against hematologic cancers.

The aforementioned rituximab, a chimeric mAb targeting CD20, marked the entry of mAbs into the treatment of hematologic malignancies (62). Initially approved by the FDA in 1997 for R/R CD20-positive B-cell NHL, rituximab’s indications have since expanded significantly. As of 2025, its FDA-approved uses include: (1) NHL – first line and R/RFL, DLBCL in combination with chemotherapy, maintenance therapy for FL after response to initial treatment; (2) CLL – in combination with chemotherapy for previously untreated or relapsed CLL; (3) autoimmune diseases: rheumatoid arthritis and granulomatosis with polyangiitis and microscopic polyangitis. Over time, newer generations of anti-CD20 antibodies emerged, such as ofatumumab, a fully human mAb that binds to a different CD20 epitope than rituximab. Ofatumumab received FDA approval in 2009 for the treatment of CLL, which was later expanded in 2014 for use in combination with chlorambucil (66). Another anti-CD20 antibody, obinutuzumab, gained FDA approval in 2013 for CLL treatment in combination with chlorambucil and in 2016 with bendamustine for R/R FL (67, 68). Daratumumab, an anti-CD38 mAb, is used in MM therapy (69), and elotuzumab, an anti-signaling lymphocytic activation molecule 7 (SLAMF7)/CDS1 mAb, received FDA approval in 2015 for R/R MM in combination with lenalidomide and dexamethasone (70). These and other antibodies have laid the foundation and set the direction for the development of novel therapies in hemato-oncology (**Table 1**).

**Table 1:** Standard mAbs, BiTEs, and CAR-T cells available for hematologic malignancies.

Active ingredient and Brand Name	Target	Indication	First-Based FDA/ EMA Registriation Date	Approval-Based Clinical Trials; Number of Participant	CR or ORR rate
Unconjugated mAbs					
Daratumumab Darzalex	CD38	R/R MM	November 2015/ April 2017	MMY3003 (NCT02076009)^4^; n = 286 (efficacy group)	ORR = 91,3%, CR = 42,3%
Elotuzumab Empliciti	SLAMF7	R/R MM	November 2015/ May 2016	ELOQUENT-2 (NCT01239797)^5^; n = 321 (efficacy group)	ORR = 79%, CR = 4%
Isatuximab Sarclisa	CD38	R/R MM	March 2020/ May 2020	ICARIA-MM (NCT02990338)^6^; n = 154 (efficacy group)	ORR = 93%, CR = 7%
Mogamulizumab Poteligeo	CCR4	R/R mycosis fungoides or Sézary syndrome	August 2018/ November 2018	Study 0761-010 (NCT01728805)^7^; n=186 (efficacy group)	ORR = 52%, CR = 2%
Obinutuzumab Gazywa	CD20	CLL	November 2013/ July 2014	CLL11 (NCT01010061)^8^; n = 238 (efficacy group)	ORR = 78,2%, CR = 28,2%
		R/R FL	February 2016/ June 2016	GADOLIN (NCT01059630)^9^; n = 155 (efficacy group)	ORR = 78,7%, CR = 15,5%
Ofatumumab Azerra	CD20	R/R CLL	October 2009/ April 2010	HuMax-CD20 (NCT00349349)^10^; n= 138	ORR = 42%, CR = 0%
		Previously untreated CLL	April 2014/ July 2014	COMPLEMENT 1 (NCT00748189)^11^; n = 221 (efficacy group)	ORR = 82%, CR = 14%
Rituximab MabThera	CD20	NHL	November 1997/ June 1998	N=166^12^	OR = 48%, CR = 4%
		CLL	February 2010/ October 2009	CLL8 (NCT00281918)^13^; n = 408 (efficacy group)	ORR = 90%, CR = 44%
Tafasitamab Monjuvi	CD19	R/R DLBCL	July 2020/ August 2021	L-MIND trial (NCT02399085)^14^; n = 80	ORR = 48%, CR = 34%
Conjugated mAbs					
Brentuximab vedotin Adcetris	CD30	Hodgkin’s lymphoma	August 2011/ October 2012	A Pivotal Open-Label Trial of Brentuximab Vedotin for Hodgkin Lymphoma (NCT00848926)^15^; n = 102	ORR = 75%, CR = 33%
		ALCL		A Phase 2 Open Label Trial of Brentuximab Vedotin (SGN-35) for Systemic Anaplastic Large Cell Lymphoma (NCT00866047)^16^; n = 58	ORR = 86%, CR = 57%
Gemtuzumab ozogamicin Mylotarg	CD33	AML	May 2000/ April 2018	Study 201/202/203^17^, n = 142 (total)	ORR = 30%, CR = 16%
Ibritumomab tiuxetan Zevalin	CD20	R/R NHL	February 2002/ January 2004	Phase I/II trial of IDEC-Y2B8 radioimmunotherapy for treatment of relapsed or refractory CD20(+) B-cell non-Hodgkin's lymphoma^18^; n=73 (efficacy group)	ORR = 80%, CR = 30%
Inotuzumab ozogamicin Besponsa	CD22	R/R ALL	August 2017/ June 2017	INO-VATE ALL (NCT01564784)^19^; n=164 (efficacy group)	ORR = 80,7%, CR = 35,8%
		R/R ALL	March 2024/ March 2023	WI203581 study (NCT02981628)^20^; n = 53	ORR = 82,5%, CR = 42%
Loncastuximab tesirine Zynlonta	CD19	R/R DLBCL NOS, DLBCL arising from low-grade lymphoma and HGBCL	April 2021/ August 2021	LOTIS-2 (NCT03589469)^21^; n = 145	ORR = 48,3%, CR = 24,1%
Polatuzumab vedotin POLIVY	CD79B	R/R DLBCL	June 2016/ January 2020	Study GO29365 (NCT02257567)^22^; n = 40 (efficacy group)	ORR = 45%; CR = 40%
BiTEs					
Blinatumomab Blincyto	CD19 x CD3	Ph(-) R/R B-ALL	December 2014/ November 2015	MT103–211 (NCT01466179)^23^; n = 189	ORR = 43%; CR = 33%
		MRD (+) BCP ALL	March 2018/ June 2018	BLAST Study, (NCT01207388)^24^; n = 116	CR = 77%
Elranatamab Elrexfio	CD3 x BCMA	R/R MM	August 2023/ December 2023	MagnetisMM-3 (NCT04649359)^25^; n = 123	ORR = 61%, CR = 35%
Epcoritamab Epkinly	CD20 x CD3E	R/R DLBCL	November 2022/ September 2023	EPCORE NHL-1 (NCT03625037)^26^; n = 157	ORR = 63,1%, CR = 38,9%
		R/R FL	June 2024/ August 2024	EPCORE NHL-2 NCT04663347^27^; n = 62	ORR = 95%, CR = 73%
Glofitamab Columvi	CD20 x CD3	R/R DLBCL, NOS or LBCL arising from FL	June 2023/ July 2023	NP30179 (NCT03075696)^28^; n = 154	ORR = 52%, CR = 39%
Mosunetuzumab Lunsumio	CD20 x CD3	R/R FL	December 2022/ June 2022	GO29781 (NCT02500407)^29^; n = 906	ORR = 72%, CR = 60%
Teclistamab Tecvayli	BCMA x CD3	R/R MM	October 2022/ August 2022	MajesTEC-1, ( NCT03145181 [Phase 1] and NCT04557098 [Phase 2])^30^, n = 165	ORR = 63%, CR = 39,4%
Talquetamab Talvey	GPRC5D x CD3	R/R MM	August 2023/ August 2023	MonumenTAL-1 (NCT03399799, NCT04634552)^31^ ^32^; n = 288	ORR = 73,6%, CR = 12,4%
Linvoseltamab-gcpt Lynozyfic	BCMA x CD3	R/R MM	February 2025/ April 2025	LINKER-MM1 (NCT03761108)^33^; n=117	ORR = 71%, CR= 50%
CAR-T					
Generic Name and Brand Name	Target	Indication	First-Based FDA/ EMA Registriation Date	Approval-Based Clinical Trials, Number of Participant	CR or ORR rate
Axicabtagene ciloleucel Yescarta	CD19	R/R PMBCL	October 2017/ August 2018	ZUMA-1 (NCT02348216)^34^, n=101	ORR = 83%, CR = 54%
		R/R DLBCL (including DLBCL arising from FL)			
		R/R FL	March 2021/ March 2022	ZUMA-5 (NCT03105336)^35^; n = 84	ORR = 92%, CR = 79%
Brexucabtagene autoleucel Tecartus	CD19	R/R MCL	July 2020/ December 2020	ZUMA-2 (NCT02601313)^36^; n = 60 (efficacy group)	ORR = 93%, CR = 67%
		R/R B-ALL	October 2021/ July 2022	ZUMA-3 (NCT02614066)^37^; n = 55	ORR = 71%, CR = 56%
Ciltacabtagene autoleucel Carvykti	BCMA	R/R MM	February 2022/ May 2022	CARTITUDE-1 (NCT03548207)^38^; n = 97	ORR = 97%, CR = 67%
Indecbtagene vicleucel Abecma	BCMA	R/R MM	March 2021/ August 2021	KarMMa, (NCT03361748)^39^; n = 128	ORR = 73%, CR = 33%
Lisocabtagene maraleucel Breyanzi	CD19	R/R LBCL (including DLBCL arising from indolent lymhoma)	February 2021/ April 2022	TRANSCEND NHL 001 (NCT02631044)^40^, n = 256	ORR = 73%, CR = 56%
		R/R HGBCL			
		R/R PMBCL			
		R/R FL (grade 3B)			
		R/R MCL	May 2024/ not yet registered	TRANSCEND-MCL (NCT02631044)^41^; n = 83 (efficacy group)	ORR = 83,1%, CR = 72,3%
		R/R CLL/SLL	March 2024/ not yet registered	TRANSCEND CLL 004 (NCT03331198)^42^; n = 65 (efficacy group)	ORR = 48%, CR = 20%
Obecabtagene autoleucel Aucatzyl	CD19	R/R B-ALL	November 2024/ not yet registered	FELIX (NCT04404660)^43^; n = 127	ORR = 78%
Tisagenlecleucel Kymriah	CD19	R/R B-ALL	August 2017/ August 2018	ELIANA, (NCT02435849)^44^; n = 75	ORR = 82% CR = 60%
		R/R DLBCL	May 2018/ August 2018	JULIET (NCT0244524)^45^; n = 93	ORR = 53%, CR = 40%
		R/R FR	May 2022/ May 2022	ELARA (NCT03568461)^46^; n = 97	CR = 69%, ORR = 86,2%,

ALCL, anaplastic large cell lymphoma; AML, acute myeloid leukemia; B-ALL, B cell acute lymphoblastic leukemia; BCP-ALL, B-cell precursor acute lymphoblastic leukemia; CLL, chronic lymphocytic leukemia; CR, complete response; DLBCL, diffuse large B cell lymphoma; FL, follicular lymphoma; HGBCL, high grade B cell lymphoma; LBCL, large B cell lymphoma; MM, multiple myeoloma; MRD, minimal residual disease; NHL, non-hodgkin's lymphoma; NOS, not otherwise specified; ORR, overall response rate; Ph, Philadelphia chromosome; PMBCL, primary mediastinal large B cell lymphoma; R/R, relapse/refractory.

However, the production of monoclonal antibodies for clinical use is constrained by several practical challenges. High production costs, difficulties in protein purification, the need for post-translational modifications, and the formation of aggregates during long-term storage limit their broader application (71, 72). both antibodies and antibody fragments often have short half-lives, requiring frequent administration or continuous infusion via *i.v.* infusion pumps, or *i.v*. drip infusions, which are burdensome for patients and increases the risk of adverse effects. These factors further escalate treatment costs (73).

In light of these challenges, IVT-mRNA technology emerges as a simple and elegant solution, offering the potential to overcome the limitations of protein-based monoclonal antibody therapies. The drawbacks of protein storage and administration can be bypassed by delivering the genetic information encoding the antibody, enabling the patient’s body to produce its own therapeutic protein (74, 75). This approach could significantly reduce production, storage, and treatment costs, thereby expanding access to advanced therapies for underserved populations and developing countries where access to costly treatments is limited or nonexistent (76–78).

The feasibility of producing fully bioactive monoclonal antibodies *in vivo* through IVT-mRNA delivery has been demonstrated in numerous studies. Unlike proteins, which require complex optimization during production, IVT-mRNA is composed of simple, repetitive building blocks, making it relatively straightforward to produce and optimize. Proteins, constructed from 20 different amino acids, exhibit vast physicochemical and biological variability, complicating their optimization. In contrast, IVT-mRNA, built from only four nucleosides, follows consistent physicochemical principles regardless of the protein it encodes. Furthermore, *in vivo* expression of IVT-mRNA encoding mAbs can be detected as early as 2 hours post-administration and can persist for hours, days, or even weeks in some tissue-targeted delivery systems, like intramuscular administration.

The concept of encoding antibodies using IVT-mRNA, rather than producing mAbs directly, was first introduced into reality in 2008 by Hoerr et al. in a patent titled “RNA-coded antibody” (EP 2101823 B1), filed by CureVac AG. This innovative approach gained scientific credibility in 2017 when Pardi et al. published a groundbreaking study demonstrating the potential of mRNA for passive immunization. Their work showed that mRNA encoding VRC01, an antibody effective against human immunodeficiency virus 1 (HIV1), could be packaged into lipid nanoparticles (LNPs) and administered intravenously. In mice, a single 30 μg dose of IVT-mRNA-LNPs led to significant antibody production in the liver, with peak levels in the bloodstream at 24 hours, gradually declining by day 11. The IVT-mRNA-LNPs encoding VRC01 outperformed traditional recombinant VRC01 mAbs in preventing HIV1 infection in a mouse model (79).

Later the same year, Thran et al. expanded on this concept, demonstrating the versatility of IVT-mRNA-based antibody delivery across various disease models. Their research highlighted the effectiveness of IVT-mRNA-LNPs encoding mAbs or camelid-derived heavy-chain antibodies (VHHs) in treating infections (e.g. rabies), toxin exposure (e.g. botulism), and cancers (e.g. lymphoma). A single injection of IVT-mRNA-LNPs generated rapid and sustained antibody responses, providing complete protection against viruses and toxins, and even eliminating tumor cells in mice. The treatment was well-tolerated, with only a brief, mild increase in cytokine levels and no evidence of liver damage or inflammation, underscoring the safety of the delivery method (80).

One prominent example was an IVTmRNA-encoded rituximab. Thran et al. engineered plasmids to produce mRNA for rituximab’s heavy (H) and light (L) chains, identifying an optimal H-to-L chain ratio 1.5:1 for effective antibody production. When administered repeatedly via LNPs in a mouse model of non-Hodgkin lymphoma, the IVT-mRNA-encoded rituximab significantly impaired tumor growth, showcasing its therapeutic potential (80).

While most studies focused on intravenous IVT-mRNA-LNPs delivery, which relies on the liver for antibody production, Tiwari et al. explored a more targeted approach for respiratory infections. They delivered IVT-mRNA encoding anti-RSV antibody (palivizumab) and VHHs directly to the lungs using intratracheal aerosols. This method proved highly effective, as RSV protection requires localized antibody presence in the lungs rather than systemic distribution. Up to 45% of lung cells produced detectable antibodies, leading to a significant reduction in RSV infection within 4 days for secreted antibodies and 7 days for membrane-anchored VHHs. Importantly, the treatment did not trigger significant lung inflammation, as cytokine levels remained stable for 24 hours after administration (81).

Collectively, these studies demonstrate the potential of IVT-mRNA-based antibody delivery as a versatile and effective alternative to traditional mAb therapies, with applications ranging from infectious diseases to cancer treatment.

### 3.2 Bispecific antibodies

#### 3.2.1 Structure and formats

The design of bsAbs originates from the structural and functional principles of natural bivalent immunoglobulins. Advances in antibody engineering have enabled the development of a wide array of bsAb formats, each tailored for specific pharmacological and clinical purposes, as no single format is universally optimal (82, 83).

BsAbs are generally classified into Fc-based and fragment-based formats, depending on the presence of the Fc region. Fc-based bsAbs, including IgG-like or IgG-appended molecules, maintain the classical IgG structure, which confers extended serum half-life and favorable tissue distribution. In contrast, fragment-based bsAbs lack the Fc domain, resulting in smaller, more modular proteins composed of at least two variable domains capable of simultaneous dual antigen binding (84).

Molecularly, bsAbs are engineered by pairing two different heavy and light chains or assembling antibody fragments with distinct antigen-binding domains. Fragment-based constructs often utilize single-chain variable fragments (scFvs)—where VH and VL domains are joined by a flexible linker—or single-domain antibodies (sdAbs or nanobodies), comprising only the VHH domain (85, 86).

Several clinically relevant fragment-based formats have been developed:

1. BiTEs^®^ (bispecific T cell engagers) consist of two scFvs, one binding a tumor antigen and the other engaging CD3 on T cells (87).
2. DARTs^®^ (dual-affinity retargeting molecules) employ a stabilized diabody framework, enhancing structural integrity and T cell activation (88).
3. TandAbs^®^ are tetravalent constructs formed by linking two diabodies, achieving bivalent binding to each antigen and extended half-life due to increased size (89).
4. BiKEs^®^ and TriKEs^®^ are NK cell engagers; TriKEs incorporate an IL-15 moiety to further stimulate NK cell proliferation and cytotoxicity (90, 91).

#### 3.2.2 Mechanism of action

**Figure 3:**
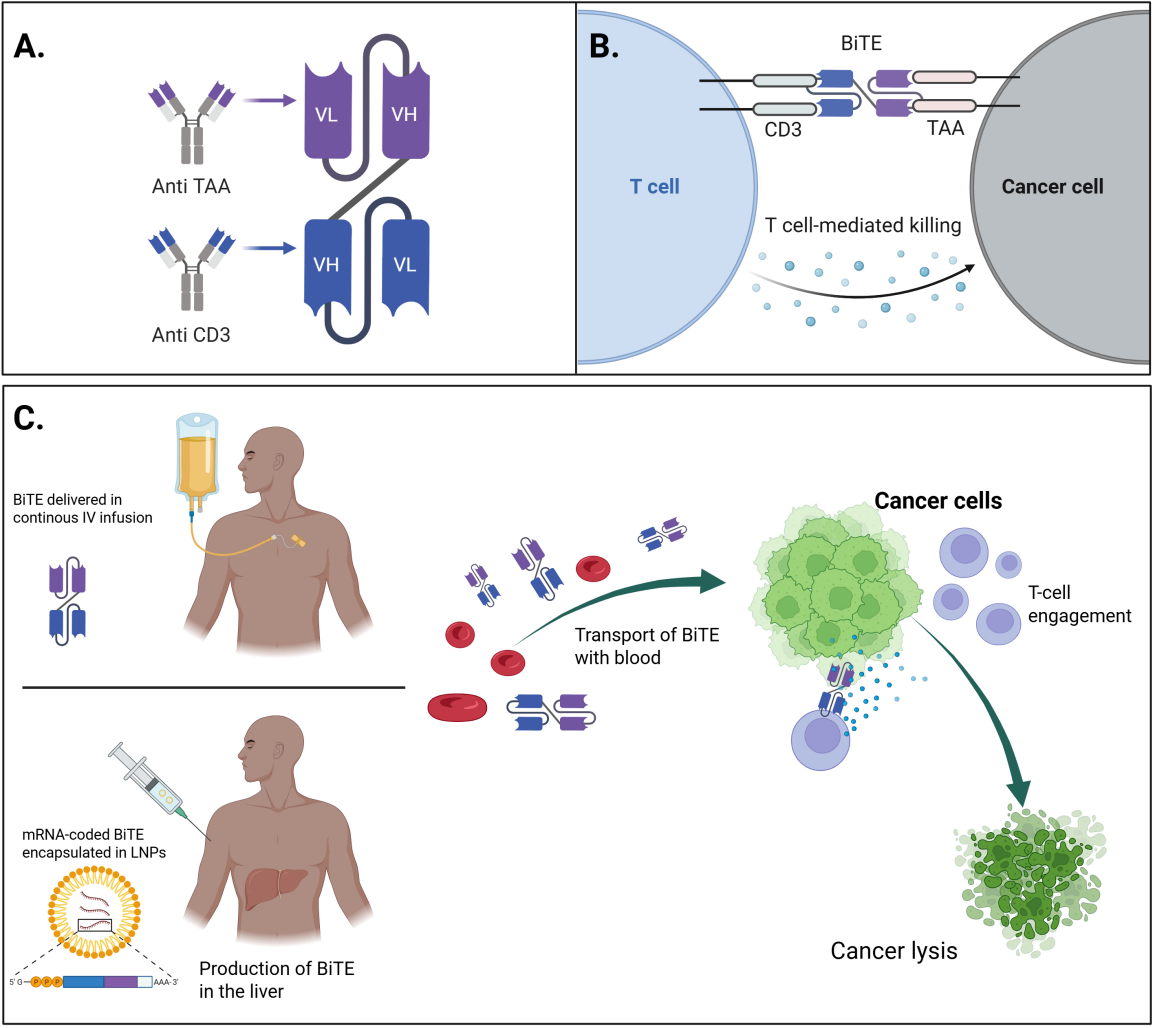
The general concept of mRNA-encoded BiTEs and their comparison with conventional therapeutics. **(A)** Schematic representation of the molecular structure of a bispecific T-cell engager (BiTE), consisting of two single-chain variable fragments (scFvs), connected by a flexible linker – one targeting a tumor-associated antigen (TAA) on cancer cells, and the other targeting CD3 on T-cells. **(B)** Mechanism of action of the BiTE molecule and comparison of delivery methods: continuous IV infusion of conventional recombinant BiTE vs mRNA-encoded BiTE encapsulated in LNPs: the BiTE simultaneously binds to the TAA on tumor cells and CD3 on T-cells, leading to the formation of a cytolytic synapse, T-cell activation, and subsequent tumor cell lysis. **(C)** Conceptual overview of mRNA-based BiTE therapy: synthetic mRNA encoding the BiTE molecule is delivered in to host cells, enabling *in situ* production and secretion of BiTE that can engage T-cells to target and eliminate cancer cells. Created with BioRender.

The mechanism of action of bsAbs can be illustrated using the fragment-based BiTE^®^ format, which functions as a T-cell engager (TCE). BiTE^®^ molecules, a key subclass of bsAbs, are composed of two scFvs linked by a flexible peptide, with a molecular weight of ~55 kDa. One scFv targets CD3ϵ on T cells, and the other recognizes a tumor-associated antigen (92, 93) major histocompatibility complex (MHC)-independent T cell activation and cytotoxicity via perforin and granzyme release. Due to their lack of Fc regions, BiTEs^®^ avoid Fc receptor-mediated off-target effects and possess enhanced tumor penetration. However, their short half-life (~2.1 hours) necessitates continuous intravenous infusion, complicating clinical use and increasing production demands (83, 94–96) (**Figure 3**) As for 2025, eight bsAbs are FDA-approved, targeting four antigens across five indications in four hematological malignancies: (A) Blinatumomab (BiTE^®^) (97, 98) targets CD19 in B-ALL, both in minimal residual disease (MRD) and R/R settings.; (B) Elranatamab (99), Teclistamab (100), Linvoseltamab (101) – target B-cell maturation antigen (BCMA) in R/R MM; (C) Talquetamab (102) - targets G protein-coupled receptor class C group 5 member D (GPRC5D) in MM; (D) Mosunetuzumab (103), Epcoritamab (104), Glofitamab (105) target CD20 in FL and DLBCL.

#### 3.2.3 Clinical challenges

The use of bsAbs and their analogs presents several challenges related to adverse effects. A comprehensive understanding of their cellular mechanisms of action and the biochemical pathways underlying these side effects is crucial for developing effective prevention and management strategies at the bedside.

##### 3.2.3.1 Modulating antibody-dependent cellular cytotoxicity

Antibody-dependent cellular cytotoxicity (ADCC) can be modulated through several strategies. Selection of IgG subclasses such as IgG2 or IgG4, which have lower affinity for Fc gamma receptors (FcγRs) compared to IgG1, can help reduce ADCC (106). Additionally, Fc-silent mutations (e.g., L234F, L235E, N297G) can prevent nonspecific immune activation via CD3/FcγR crosslinking, enhancing T cell recruitment to the tumor microenvironment (TME) and limiting complement activation. Fc silencing is particularly advantageous for bsAbs focused on immune modulation, such as TCEs and immune checkpoint-targeting bsAbs (107). Conversely, enhancing FcγR interactions can potentiate immune activation for bsAbs that block tumor-promoting pathways (e.g., epidermal growth factor (EGFR) or human epidermal growth factor receptor 2 (HER2)), boosting antitumor efficacy (108). Reducing or eliminating core fucose in Fc N-glycans increases IgG1-FcγRIIIa binding, further enhancing ADCC, as demonstrated in monoclonal antibodies like trastuzumab and bsAbs such as amivantamab (EGFR × cMET DuoBody) (109, 110).

##### 3.2.3.2 Pharmacokinetics and biodistribution

Modifications to bsAb molecular structures also influence biodistribution and pharmacokinetics (PK). BsAbs are recycled *via* the neonatal Fc receptor (FcRn) pathway, which protects IgGs from degradation by binding them in acidic endosomes and releasing them back into circulation at neutral pH, thus prolonging half-life (111). FcRn binding site mutations (e.g., Q311R, M428L) can enhance dissociation at pH 7.4, improving serum persistence and efficacy. IgG subclass choice also impacts half-life (112, 113). Fragment-based bsAbs, although smaller and better at penetrating the TME, exhibit shorter half-lives and faster clearance, necessitating frequent dosing or continuous infusion (83). Strategies to extend half-life include fusion to human serum albumin (half-life ~19 days) or incorporation of Fc domains into fragment-based bsAbs (e.g., HLE-BiTEs^®^, DART^®^-Fc formats, NCT05740666) (114, 115). Subcutaneous administration, as explored in blinatumomab (NCT04521231), is another method that can prolong drug exposure by mimicking continuous infusion.

##### 3.2.3.3 On-target, off-tumor toxicity

BsAbs are also associated with unique toxicities, notably on-target, off-tumor effects. Dual targeting approaches may inadvertently affect healthy tissues expressing the target antigen (116). Designing the second binding arm to recognize tumor-specific antigens can shift activity toward malignant cells. For instance, 4-1BB-targeting bsAbs minimize hepatotoxicity by requiring TME-specific activation. ABL503 (PD1 × 4-1BB, IgG-scFv2) demonstrated reduced liver toxicity and superior antitumor activity compared to mAb combinations in preclinical models (117–119). TG-1801 (CD47 × CD19, κλ body) combines a high-affinity CD19 arm with a low-affinity CD47 arm, selectively targeting malignant B cells overexpressing CD47, while sparing normal cells. Early clinical results show promising safety and efficacy (120). Another approach involves protease-cleavable masking of bsAbs, allowing activation specifically within hypoxic, protease-rich TME (121). TAK-280 (CD3 × B7H3, COBRA TCE), currently in phase 1 trials for metastatic solid tumors, exemplifies this strategy.

##### 3.2.3.4 Effects on regulatory T-cells and immune memory

The impact of TCEs on regulatory T cells (Tregs) remains unclear, though there is a concern that Tregs may suppress TCE activity. TCEs activate T cells, induce T cell margination (TCM) and proliferation, reshape the TME, and trigger cytokine release, which attracts additional immune cells (94). Although originally believed to be MHC-independent, TCEs may exhibit enhanced T cell expansion via peptide-MHC class I interactions, as seen in CD3 × BCMA bsAbs for multiple myeloma (122). Their effect on long-term T cell memory, however, remains under investigation. Novel TCE designs are emerging, including LAVA-051 (Vy9Vδ2 T cell engager × CD1d) for leukemia/myeloma (123). NK cell-directed bsAbs, such as BiKEs^®^ (e.g., AFM13: CD30 × CD16A; RO7297089: BCMA × CD16A), are also under development (124, 125).

##### 3.2.3.5 Cytokine release syndrome

Cytokine release syndrome (CRS) is a potentially severe, though rare, complication of TCE therapy, characterized by excessive secretion of inflammatory cytokines (IL-6, IFN-γ, TNF-α). Severe CRS can lead to hypotension, capillary leak syndrome, and multi-organ failure. While all-grade CRS is common (e.g., 75-79% with talquetamab), grade ≥3 events are rare (0-3%). CRS onset varies by therapy: minutes to hours for rituximab, days to weeks for CAR-T cells, and typically within 48 hours of first bsAb dose (102, 126, 127).

##### 3.2.3.6 Immune effector cell-associated neurotoxicity syndrome

Immune effector cell-associated neurotoxicity syndrome (ICANS) may co-occur with CRS but involves distinct mechanisms. Its pathogenesis involves more directly the central nervous system (CNS), disrupting the brain-blood barrier (BBB) via the CNS endothelial activation. Key cytokines involve IL-1 and IL-6. Triggered by excessive immune activation, ICANS presents with tremors, aphasia, apraxia, and in severe cases, seizures or coma. Risk factors include small molecule size, TCE mechanisms, and tumor antigen expression in neural tissue (128–130).

##### 3.2.3.7 Infusion-related reactions

Infusion-related reactions (IRRs), including chills, dyspnea, flushing, and nausea, typically arise within 10 minutes to 4 hours of infusion onset. IRRs are Type B (bizarre) reactions, unpredictable and unrelated to dose or pharmacology. They are more common with mAbs than bsAbs but increase with bsAbs targeting dual signaling pathways or immune checkpoints, as seen with MCLA-129 (anti-EGFR/MET, 90% IRRs) and amivantamab (67%) (131–133).

##### 3.2.3.8 Infection risk and immunosuppression

Patients with hematologic malignancies often experience immunosuppression due to disease or prior treatments (e.g., cytopenias, hypogammaglobulinemia, CAR-T therapy, bone marrow transplant), increasing susceptibility to opportunistic infections (fungi, CMV, Gram-negative bacteria). BsAb-induced lymphocyte activation and on-target off-tumor effects (e.g., plasma cell aplasia from BCMA/GPRC5D/FcRH5-targeting bsAbs), as well as immunosuppressive agents used for CRS management, may further compromise immunity (134–136).

##### 3.2.3.9 Resistance mechanisms

Resistance to bsAbs can arise through multiple mechanisms. Immune checkpoint upregulation, such as PD-L1 expression, reduces TCE efficacy. For example, AMG 330 (CD3 × CD33 BiTE^®^) showed reduced cytotoxicity in AML due to PD-L1 induction. PD-1/PD-L1 blockade restored TCE activity, increasing AML lysis, T cell proliferation, and IFN-γ secretion (137, 138). In B-NHL, low baseline PD-1 expression correlated with response to glofitamab (CD3 × CD20) (139), while combining odronextamab (CD3 × CD20) with anti-PD1 antibodies enhanced antitumor effects (140). These findings suggest that immune checkpoint upregulation is a reversible resistance mechanism, and dual TCE-ICI targeting may improve outcomes. Several trials (e.g., NCT02879695, NCT03340766, NCT03512405) are investigating this approach.

Antigen loss also contributes to resistance. CD19 loss occurs in 6-30% of R/R B-ALL cases, mainly via disrupted membrane trafficking (141). While alternative targets like CD20 or CD22 remain, antigen loss also affects efficacy of therapeutics like glofitamab. Strategies to overcome this obstacle include dual-antigen targeting (e.g., blinatumomab + inotuzumab, NCT03739814), or preventing antigen loss through epigenetic modulation. In multiple myeloma, BCMA downregulation post-TCE therapy, as observed with AMG 420 (CD3 × BCMA), leads to resistance (142). BCMA loss also limits CAR-T efficacy (143).

BsAb therapy introduces challenges, particularly in sequencing with CAR T-cell therapies, especially when targeting the same antigen. In B-ALL, CD19 antigen loss following blinatumomab may compromise subsequent CD19-directed CAR-T therapy (144, 145), although early response to blinatumomab may predict CAR-T success (146). Conversely, small studies suggest blinatumomab remains effective post-CAR-T (147), though further data are required. In MM, bsAbs are being explored as bridging therapies prior to CAR-T to enhance T cell expansion and improve CAR-T persistence. However, due to limited clinical evidence, these decisions remain largely individualized (100, 148, 149). Notably, no curative potential has yet been demonstrated for MM. In DLBCL, the issue of antigen escape is minimized as CAR-T targets CD19 and bsAbs target CD20. Emerging data suggest that prior or subsequent use of either modality does not significantly impair efficacy (105, 150).

Impaired IFN-γ signaling, particularly through JAK2 downmodulation, reduces tumor sensitivity to T cell-mediated killing, as reported in HER2-targeting bsAbs (151). Whether this resistance extends to non-HER2 bsAbs or hematologic malignancies remains unclear.

ADAs may target bsAb variable regions, blocking antigen binding, altering pharmacokinetics, or inducing immune toxicities. ADA development is influenced by bsAb immunogenicity (e.g., foreign sequences, aggregation-prone motifs), administration route, and patient immune status (152). Subcutaneous delivery poses higher ADA risk due to dendritic cell activation in the skin, making IV delivery preferable in most cases (153).

#### 3.2.4 mRNA-enabled therapies

Unlike recombinant proteins, IVT-mRNA enables *in situ* production of therapeutic bsAbs following a single administration. This results in transient, self-limited expression, eliminating the logistical burden of continuous infusion required for short-lived BiTE^®^ formats and reducing pharmacokinetic extremes that contribute to toxicity (92, 154). The transient expression also enables step-up or fractionated dosing strategies to mitigate CRS and IRRs (102, 127) without the production burdens inherent to protein-based therapies.

By encoding Fc-silent or Fc-free- bsAbs, IVT-mRNA constructs avoid Fcγ receptor–mediated off-target effects and complement activation, addressing ADCC modulation strategies such as Fc mutations (L234F, L235E, N297G) used to reduce toxicities (106, 107). This strategy preserves high local tumor efficacy without systemic immunologic collateral damage.

mRNA-coded constructs can incorporate protease-activated masking, similar to COBRA or TAK-280 formats, ensuring activation only within the protease-rich tumor microenvironment and thereby minimizing systemic or hepatic toxicities related to on-target - off-tumor binding (121).

The versatility of IVT-mRNA platforms further supports multi-specific or costimulatory formats. For example, mRNA can co-encode tri-specific agents targeting simultaneously CD38, CD3, and CD28 or combine TCE with immune checkpoint blockade (PD-1/PD-L1 or 4-1BB), confronting resistance mechanisms such as antigen loss, checkpoint upregulation, and lack of memory T cell generation. These modular combinations, previously shown to restore BiTE^®^ efficacy when paired with immune checkpoint inhibitors (137, 138), can now be delivered via a single IVT-mRNA platform. Preclinical data validating Fc-free bsAb IVT-mRNAs, such as EGFR × CD3 LiTE and PD-L1 × 4-1BB Albu-LiTCo, confirm this approach’s feasibility (155).

Pharmacokinetically, IVT-mRNA-encoded antibodies exhibit a controlled, depot-like profile. LNPs enable efficient uptake and endosomal escape, while no genome integration ensures safety (79, 80, 155). Subcutaneous or intramuscular delivery, particularly in engineered depot formulations, mimics continuous infusion without sustained high serum peaks, reducing CRS and IRRs risk (93, 94).

Regarding cytokine release syndrome, IVT-mRNA-encoded bispecific molecules have demonstrated favorable safety profiles. In the preclinical CLDN6 mRNA-BiTE^®^ studies, only low, transient cytokine elevations were detected, with no evidence of systemic CRS in mice and cynomolgus models (154). In humans, the BNT14201 Phase I/II trial of an mRNA-LNP - encoded CLDN6 × CD3 bispecific reported mild cytokine elevations in 22% of patients, with only one case of grade 3 CRS among 65 patients - an acceptable safety profile compared to protein-based bsAbs (154)(Stadler et al., 2024; OncoDaily Jun 1 2025).

Beyond systemic delivery, local IVT-mRNA strategies, such as intra-tumoral injection of LNPs encoding IL-12, IFN-α, and IL-7, generate robust antitumor immunity and depot-like expression while minimizing systemic exposure - offering potential to avoid IRRs, ICANS, and infections associated with systemic immunomodulation (156).

Furthermore, the manufacturing advantages of IVT-mRNA are significant. Rapid, cell-free synthesis bypasses costly protein expression, folding, glycosylation, and cold-chain transportation, facilitating scalable production - even for personalized or regionally targeted therapies (71, 76).

Finally, IVT-mRNA’s transient expression profile helps minimize long-term immunosuppression and infection risk by allowing recovery of normal B and T cell populations post-treatment (134). It also avoids persistent ADA responses that are more likely with protein therapeutics or prolonged exposure of fragment-based bsAbs (153).

In summary, IVT-mRNA enabled bispecific therapies seem to directly address each clinical challenge of bsAbs: by modulating Fc biology, controlling pharmacokinetics, reducing toxicities including CRS/ICANS, optimizing dosing strategies, preventing resistance, easing manufacturing burdens, and preserving immune competence. The BNT142 program serves as a proof of concept that these advantages can be realized safely in humans. Continued clinical development and combination studies will clarify their long-term potential in hematologic and solid tumor indications.

### 3.3 CAR-T cells

In parallel with the rapid development of IVT-mRNA technology and its therapeutic applications, CAR-T cells have revolutionized the treatment of R/R hematological malignancies. It is associated with impressive response rates, ranging up to 54% for large B-cell lymphoma (LBCL) (157) and up to 93% for B-ALL (158). However, many patients relapse, with various mechanisms responsible for the failure. Moreover, safety concerns regarding transgene integration or uncontrolled proliferation are raised. On top of that, the manufacturing cost is high and often makes the therapy unaffordable.

The challenges mentioned above are somewhat attributable to the manufacturing process and the technology itself. Currently, the CAR-T product is based on autologous (or allogeneic in some studies) cells which are *ex vivo* transduced with CAR-coding viral DNA. Importantly, viral DNA is incorporated into the genome of T-cells. As a result, CAR-T cell therapy is dependent on a single batch of lymphocytes that are programmed to constantly target specific antigens and have limited *in vivo* persistence.

**Figure 4:**
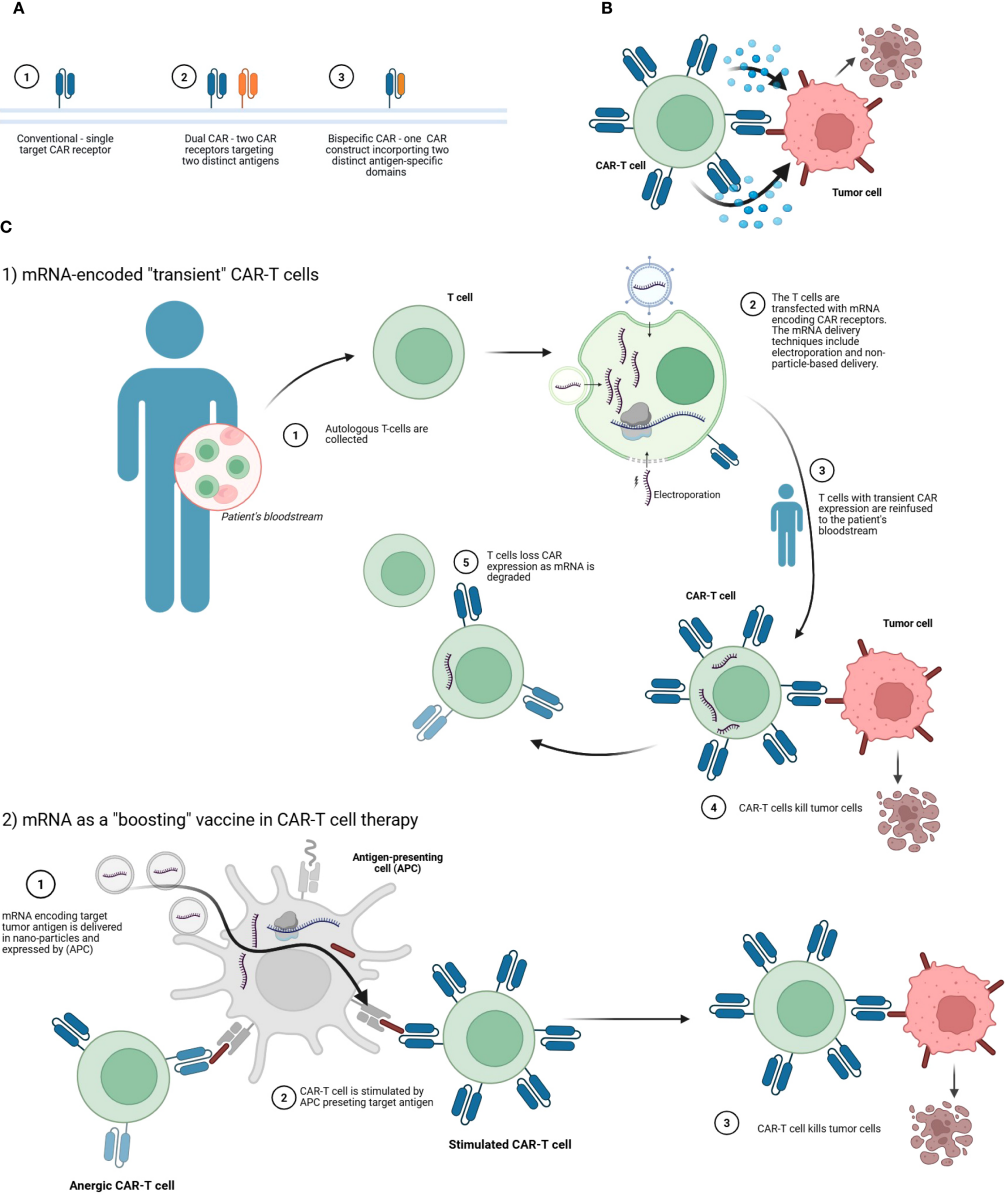
Strategies involving mRNA-engineered CAR-T cell therapies. **(A)** Comparison of CAR-T receptor configurations. The panel illustrates three structural formats: (1) a conventional CAR with a single target specificity, (2) a dual CAR system comprising two separate receptors for two distinct tumor-associated antigens, and (3) a bispecific CAR consisting of a single construct that integrates two antigen-recognition domains targeting different antigens. These configurations are designed to enhance tumor recognition and reduce antigen escape. **(B)** Mechanisms of tumor cell elimination by CAR-T cells. The CAR-T cell engages the tumor cell *via* its chimeric antigen receptor, leading to immune synapse formation, cytokine release, and tumor cell lysis. **(C)** mRNA-based applications in CAR-T cell therapy. C.1. Transient CAR expression *via* mRNA transfection. Autologous T-cells are collected from the patient’s blood and transfected *ex vivo* with mRNA encoding CAR receptors using methods such as electroporation. The resulting CAR-T cells, expressing the receptor transiently, are reinfused into the patient. These modified T-cells can then recognize and kill tumor cells. Over time, CAR expression wanes as the mRNA degrades, providing a controlled and reversible therapeutic effect. C.2. mRNA as a vaccine-like to boost CAR-T responses. mRNA encoding the tumor-associated antigen is delivered in nanoparticles and expressed by antigen-presenting cells (APCs). This stimulates anergic or suboptimally active CAR-T cells by presenting the target antigen in a costimulatory context, restoring their effector functions. The reactivated CAR-T cells then eliminate tumor cells more effectively. Created with BioRender.

The incorporation of IVT-mRNA technology into CAR-T therapy creates an opportunity to bypass these limitations and provides new solutions for more flexible therapy. (**Figure 4**) These stem from the transient expression of IVT-mRNA-encoded CARs. Currently evaluated mRNA-based approaches to CAR-T cell therapy include the following:

1. *ex vivo* manufacturing of IVT-mRNA CAR-T cells,
2. *in vivo* generation of IVT-mRNA CAR-T cells.

By default, all these approaches rely on transient CAR-T cells that are capable of time-limited tumor killing. The main advantage is that the IVT-mRNA-based approach could mitigate long-term adverse events such as B cell aplasia and pancytopenia. Moreover, with the use of IVT-mRNA there is no risk of unwanted genome integration of CAR-encoding genes (159).

However, transient expression may necessitate repeated infusions of *ex vivo*-manufactured CAR-T cells or IVT-mRNA boosters, when the tumor is not cleared (159). This may lead to an increased financial burden. Nevertheless, each IVT-mRNA-based approach offers some advantages over conventional CAR-T cells, but at the same time each has its drawbacks.

#### 3.3.1 *Ex vivo* manufacturing of mRNA CAR T cells

The first approach, namely *ex vivo* manufacturing of IVT-mRNA CAR-T cells, is the most similar to the conventional DNA-based approach as the cells must be collected from the donor and processed in the laboratory. In the production process, mRNA is delivered to T-cells using either electroporation techniques or IVT-mRNA delivery carriers such as LNPs (160). Electroporation is a relatively straightforward and therefore the most common technique for manufacturing *ex vivo* IVT-mRNA CAR-T cells (160, 161). However, it is associated with poor transfection rates and is toxic to T-cell (162) Combined with the IVT-mRNA instability and need for thorough purification, *ex vivo* manufacturing of IVT-mRNA CAR-T cells is costly and labor-intensive (160). In the field of hematology, the discussed approach has been implemented in the NCT03448978 trial investigating IVT-mRNA CAR-T cells targeting BCMA in MM (163, 164). However, only data regarding one patient who achieved a very good partial response (VGPR) have been published so far (163).

#### 3.3.2 *In vivo* generation of mRNA CAR T-cells

The second approach, namely *in vivo* production of IVT-mRNA CAR-T cells, is more appealing as it allows to shorten the waiting time and could be administered off-the-shelf. The most common choice of *in vivo* IVT-mRNA delivery are IVT-mRNA nanocarriers targeting specific antigens (160). Parayath et al. conducted a seminal study on the production of IVT-mRNA CAR-T cells *in vivo* (165). In a mouse model of lymphoma (mice inoculated with CD19^+^ Raji cells), they proved that lymphocyte-targeted IVT-mRNA nanoparticles could deliver IVT-mRNA to T-cells and achieve comparable responses to conventional DNA-based CAR-T cells manufactured *ex vivo*. Crucially, the IVT-mRNA CAR-T cells did not contribute to acute systemic toxicities. However, this approach required repeated infusions of IVT-mRNA carrier nanoparticles. Unfortunately, the authors emphasize that the development of effective IVT-mRNA CAR-programming nanoparticles is very complex and therefore could affect the clinical application of this approach (165). Both *in vivo* and *ex vivo* CAR-T approaches face a fundamental limitation: they depend on the patient’s endogenous T-cell function, which is often compromised by prior therapies. While *ex vivo* methods allow for T-cell selection and expansion, neither strategy can fully overcome poor lymphocyte quality in heavily pretreated patients, highlighting the need for alternative solutions like immune reconstruction therapies (166).

Finally, IVT-mRNA technology can be applied to CAR-T cell therapy by delivering IVT-mRNA encoding a target tumor antigen in a vaccine-like manner to stimulate CAR-T cells *in vivo*. In this approach, IVT-mRNA-LNPs are taken up by various cells, primarily macrophages and other antigen-presenting cells (APCs), which then express the encoded membrane-bound tumor antigen. This antigen – often a conformational epitope of the native protein – can engage and activate CAR-T cells when they encounter the APCs or other expressing cells. A seminal phase-1 study by Mackensen et al. demonstrated that IVT-mRNA vaccine-like boosting could enhance CAR-T cell expansion *in vivo* (167). However, the precise location of this stimulation – whether in lymphoid organs (e.g. lymph nodes) or peripheral tissues – remains unclear. Importantly, the study focused on solid tumor, and given these promising results, similar investigations in hematological malignances are highly anticipated.

## 4 Clinical translations and challenges

Building on the mechanistic insights and preclinical innovations described in previous sections, this section consolidates the current clinical landscape, focusing on the translation of IVT-mRNA-based approaches into hematologic oncology trials. As summarized in **Table 2**, early mRNA-based CAR-T and bispecific trials demonstrate feasibility and manageable safety but limited persistence and efficacy compared with conventional platforms. Clinical data are categorized and analyzed across the principal modalities - CAR-T cells and bispecific antibodies - highlighting their potential, limitations, and lessons for future development.

**Table 2:** Overview of mRNA-based and conventional adoptive cell therapies and bispecific antibodies in clinical development or practice.

Platform	Product / Trial (NCT)	Target	Malignancy	Phase	Group Size (n)	Key Outcomes (ORR/CR etc.)	Main Toxicities (with frequencies)
mRNA-engineered CAR-T (ex vivo)	Descartes-08^47^, NCT03448978	BCMA	Multiple Myeloma	I	<20	Case report: VGPR; early activity	Mostly grade 1–2 AEs; no ≥G3 CRS/neurotoxicity reported
mRNA-engineered CAR-T (ex vivo)	NCT02277522 (adult) / NCT02624258 (pediatric)^48^	CD19	Hodgkin Lymphoma	I	<20	Transient responses, no durable remissions	no persistent severe adverse events
mRNA-engineered CAR-T (ex vivo)	NCT02623582^49^	CD123	AML	I	7	No sustained responses	Mild/moderate CRS; no severe neurotoxicity; early cytopenias
mRNA-encoded bispecific (in vivo)	NCT05262530^50^	CLDN6×CD3	CLDN6+ solid tumors	I/II	65	Early activity (DCR/PR in CLDN6+)	TRAEs 63%; ≥G3 TRAEs 23%; CRS 22% (1 ≥G3); AST/ALT↑19% (12% ≥G3)
Conventional CAR-T (viral)	ZUMA-1 — Axicabtagene ciloleucel (axi-cel) NCT02348216^51^	CD19	LBCL	II	101 (pivotal); >2000 real-world	ORR ~83%, CR ~58%	CRS all-grade ~93%; CRS ≥G3 ~11%; ICANS all-grade ~42%; ICANS ≥G3 ~32%; neutropenia ≥G3 ~24%; thrombocytopenia ≥G3 ~43%; infections ~32%
Conventional CAR-T (viral)	JULIET— Tisagenlecleucel (tisa-cel) NCT02445248^52^	CD19	DLBCL	II	115	ORR ~52%, CR ~40%	CRS all-grade ~58%; CRS ≥G3 ~6%; ICANS ≥G3 ~12%; cytopenias common; infections ~20%
Conventional bispecific (BiTE)	Blinatumomab NCT02003222^53^	CD19×CD3	B-ALL	III	224	ORR 81%, CR 81%	CRS ~2%; neurotoxicity 52%, neutropenia, thrombocytopenia
Conventional bispecific (IgG-like HLE)	Teclistamab (MajesTEC-1) NCT04557098^54^	BCMA×CD3	Multiple Myeloma	I/II	165	ORR ~63% (30 mo follow-up)	CRS 72% (≥G3 0.6%); neurotoxicity 57%; ICANS 6% (≥G3 ~2.4%); pneumonia 15%; sepsis 6%
Conventional bispecific (IgG-like HLE)	Talquetamab (MonumenTAL-1) NCT03399799^55^	GPRC5D×CD3	Multiple Myeloma	I/II	288	ORR ~70% in heavily pretreated MM	CRS ~75% (≥G3 <1%); ICANS ~10% (rare ≥G3); skin/nail/taste toxicities ~60–70%
Conventional bispecific (IgG-like HLE)	Epcoritamab (EPCORE NHL-1) NCT03625037^56^	CD20×CD3	DLBCL	I/II	157	ORR ~63%, CR ~39%	CRS 49% (≥G3 ~2%); ICANS ~6% (rare ≥G3); neutropenia ≥G3 ~30%
Conventional bispecific (IgG-like HLE)	Glofitamab (NP30179) NCT03075696^57^	CD20×CD3	DLBCL	I/II	155	ORR ~52%, CR ~39%	CRS 63% (≥G3 ~4%); ICANS ~3% (rare ≥G3); cytopenias frequent

The table summarizes key early-phase trials of mRNA-engineered CAR-T cells (ex vivo) and mRNA-encoded bispecifics (in vivo), alongside pivotal studies of conventional viral CAR-T therapies and IgG-like bispecific antibodies. Reported outcomes include objective response rates (ORR), complete response (CR) rates, and selected toxicities such as cytokine release syndrome (CRS), neurotoxicity (ICANS), cytopenias, and infections. mRNA-based approaches demonstrate transient activity with favorable safety in early trials, while conventional CAR-Ts and bispecific antibodies show established efficacy with characteristic toxicity profiles.

### 4.1 mRNA-engineered CAR-T cells

While viral vector-based CAR-T cells have transformed the treatment landscape for B-ALL, DLBCL, and MCL, their limitations in cost, safety, and long-term antigen persistence have driven exploration of IVT-mRNA-based CAR-T platforms. As described previously, IVT-mRNA enables transient CAR expression, mitigating risks of genomic integration and prolonged immune activation (160).

Clinical data, however, remain limited. In MM, the *Descartes-08* program (NCT03448978 (163),) evaluated *ex vivo*-transfected anti-BCMA CAR-T cells in a small Phase I cohort (<20 patients). A case report documented a very good partial response (VGPR), suggesting early activity, although CAR expression was transient. In HL, two Phase I studies of anti-CD19 mRNA CAR-T cells (NCT02277522 in adults; NCT02624258 in pediatric patients) reported no unexpected grade ≥3 toxicities, but responses were transient and no durable remissions were achieved (168). Similarly, an anti-CD123 IVT-mRNA CAR-T program in acute myeloid leukemia (NCT02623582) enrolled seven patients but failed to generate sustained responses; the trial was terminated early due to manufacturing issues and lack of efficacy (169).

These early trials highlight the feasibility and short-term safety of IVT-mRNA CAR-T products, but underscore persistent challenges with manufacturing reliability, CAR persistence, and clinical efficacy - particularly in heavily pretreated or myeloid malignancy settings. Novel strategies, including *in vivo* CAR-T programming (165) and IVT-mRNA vaccine boosters for CAR-T expansion (167), warrant further investigation to overcome these barriers.

By contrast, conventional viral vector-engineered CAR-T therapies have demonstrated robust and durable activity in large B-cell lymphomas. In the pivotal *ZUMA-1* study of axicabtagene ciloleucel (axi-cel), the objective response rate (ORR) was ~83% with a complete remission (CR) rate of ~58%, findings later reproduced in >2,000 real-world patients (157). Likewise, the *JULIET* trial of tisagenlecleucel (tisa-cel) in diffuse large B-cell lymphoma reported an ORR of ~52% and a CR rate of ~40% (170). These outcomes underscore the therapeutic gap between transient mRNA CAR-T products and durable viral CAR-T platforms.

### 4.2 mRNA-encoded bispecific antibodies

The success of bispecific antibodies (bsAbs) such as blinatumomab and teclistamab has paved the way for exploring IVT-mRNA as a means of *in vivo* bsAb production, potentially overcoming the pharmacokinetic and production constraints of protein-based therapies. The BNT142 program (Phase I/II) tested a lipid nanoparticle-encapsulated IVT-mRNA encoding a CLDN6×CD3 bispecific in patients with CLDN6-positive solid tumors. While outside hematology, the trial reported encouraging safety—only one of 65 patients experienced grade 3 CRS, and cytokine elevations were transient in ~22% of patients (154). These findings support the feasibility of IVT-mRNA-encoded bispecifics, with the potential to achieve controlled local activity and reduced systemic toxicity through stepwise dosing or protease-activated masking.

In hematologic malignancies, conventional bispecifics have set a high efficacy benchmark. The CD19×CD3 BiTE blinatumomab achieved an ORR and CR rate of 81% in a Phase III trial in B-ALL but requires continuous infusion due to its short half-life (171). Newer IgG-like half-life–extended (HLE) bispecifics combine potent activity with more convenient administration. Teclistamab (BCMA×CD3, *MajesTEC-1*) demonstrated an ORR of ~63% with durable responses beyond 30 months in R/R MM (100). Talquetamab (GPRC5D×CD3, *MonumenTAL-1*) produced an ORR of ~70% in heavily pretreated myeloma (172). In aggressive B-cell lymphomas, epcoritamab (CD20×CD3, *EPCORE NHL-1*) and glofitamab (NP30179) achieved ORRs of ~63% and ~52%, with CR rates of ~39% each (104, 105).

## 5 Future perspectives

### 5.1 mRNA, bioinformatics and artificial intelligence

Therapeutic IVT-mRNA requires optimal design to ensure stability, efficient translation, and targeted activity. Recent progress in bioinformatics and artificial intelligence (AI) has significantly advanced the prediction and optimization of IVT-mRNA therapeutics, and their integration is emerging as a key driver of innovation. The growing demand for optimized IVT-mRNA highlights the indispensable role of computational tools in therapeutic development.

Traditionally, IVT-mRNA sequence optimization has relied on foundational bioinformatics approaches. For secondary structure prediction, tools such as RNAfold, mFold, and IPKnot are widely used to identify conformations that enhance translational efficiency. Complementary to this, molecular dynamics simulations implemented in platforms including GROMACS, NAMD, AMBER, and CHARMM enable the examination of IVT-mRNA three-dimensional architecture and folding dynamics (173–175). Codon optimization represents another critical layer of design, with algorithms such as GeneOptimizer and JCAT tailoring coding sequences to host-specific codon usage and tRNA availability, thereby maximizing protein output (176, 177).

Delivery systems, LNPs, also benefit from in silico optimization. Recent studies have employed molecular dynamics simulations to investigate lipid self-assembly and protonation behavior of ionizable lipids, while high-throughput screening and platforms such as NANOdesign, POLYVIEW-3D, pyMOL, and COMSOL NanoAssembler have been used to optimize PEG-lipid ratios, improving stability, biodistribution, and therapeutic index (178–180). These insights are directly relevant to preclinical hematology and oncology applications: optimized LNP formulations have successfully delivered nucleic acids in CML models, reducing leukemic burden with minimal toxicity (181, 182), while novel ionizable lipids have enhanced IVT-mRNA retention at injection sites and reduced off-target accumulation in the liver, improving the safety and efficacy of tumor vaccines (182–184).

Understanding IVT-mRNA folding and function requires predictive models that capture both thermodynamic and kinetic parameters. Tools such as RNAfold, mFold, and IPKnot anticipate higher-order structures using thermodynamic and entropic criteria (185–187). Deep learning models are increasingly able to predict IVT-mRNA folding pathways and structural conformations, complementing experimental techniques such as NMR spectroscopy, cryo-electron microscopy, and chemical probing, which provide high-resolution validation but are more resource-intensive (188). Beyond secondary structure, IVT-mRNA modifications such as N6-methyladenosine (m^6^A) exert critical regulatory influence. In hematopoietic malignancies, altered m^6^A landscapes impact IVT-mRNA stability, translation, and splicing, representing both a biological challenge and a therapeutic opportunity (189, 190).

AI and machine learning are becoming integral to IVT-mRNA therapeutic development. General algorithms such as XGBoost, Graph Convolutional Networks (GCNs), and deep neural networks (DNNs) are methodological cornerstones. Frameworks such as TensorFlow and PyTorch enable DNNs to refine vaccine design using *in vivo* data (191–195). In hematology, machine learning has been applied to predict immunogenic epitopes and optimize LNP formulations for hematopoietic targeting. These approaches have accelerated candidate selection, though fully end-to-end demonstrations of deep learning-designed AML IVTmRNA vaccines with *in-vivo* validation are still limited in the published literature (183, 196). Most recently, GEMORNA, a generative AI platform, has demonstrated the ability to design novel linear and circular RNA sequences with markedly improved expression, durability, and *in vivo* immunogenicity compared to existing benchmarks (197).

Several breakthroughs illustrate the translational relevance of computational design. The LinearDesign algorithm, which simultaneously optimizes codon usage and secondary structure, has been experimentally validated to improve IVT-mRNA half-life, protein expression, and immunogenicity *in vivo* (198). Coarse-grained simulations have provided valuable insights into the self-assembly of LNPs, revealing how lipid composition and pH influence LNP morphology and IVT-mRNA release. These simulations offer predictive frameworks that can guide the design of LNPs with enhanced *in vivo* delivery efficiency (199). AI-powered tools such as gRNAde predict mRNA 2D and 3D conformations with high accuracy, while Wong et al. (2024) introduced a structural AI platform that generates RNA sequences based on target 3D architectures, significantly reducing experimental costs (200, 201). Collaborative initiatives such as RNA-Puzzles and CASP15 continue to benchmark predictive accuracy across the field (202, 203).

Taken together, these advances demonstrate that bioinformatics and AI are no longer speculative additions but validated tools in IVT-mRNA therapeutic design. Their role is particularly evident in hematology, where codon usage studies, RNA modification research, and LNP delivery improvements are supported by preclinical data in leukemia and lymphoma. As these computational frameworks continue to integrate with experimental validation, they are poised to accelerate the development of next-generation IVT-mRNA therapies in oncology and hematology.

### 5.2 Large-scale population studies and broader accessibility of mRNA

Hematologic malignancies represent a heterogeneous group of cancers, with genetic mutations playing a central role in their classification. The dynamic and diverse nature of these diseases necessitates a deeper understanding of their genomic and environmental determinants to enable early risk detection and personalized therapies.

A persistent challenge is the lack of diversity in clinical trials. For example, teclistamab/talquetamab trials included only 10–14% Black participants, while Hispanic representation was unreported (102). Similarly, elranatamab trials featured 20% Black participants, with no Hispanic data (99). Many BsAb trials, including those for mosunetuzumab, epcoritamab, and glofitamab, omitted racial/ethnic breakdowns (103, 105, 204). Disparities are stark: non-Hispanic Black individuals face twice the risk of MM yet have limited trial access (205).

In Europe, aging populations and rising hematologic cancer incidence strain healthcare systems, underscoring the need for systemic innovations. A 2023 study analyzing 30 years of global data revealed 1.34 million new cases in 2019, with declining mortality rates reflecting therapeutic advances (8). However, data gaps persist in low-income regions. Gender disparities were evident, with higher incidence among males (MM: 1.4:1; NHL: 1.6:1). Advances in new generation sequencing (NGS) and flow cytometry have refined cancer subtyping (e.g., breakpoint cluster region – Abelson murine leukemia viral oncogene homolog 1 (BCR-ABL1) detection in AML), though diagnostic reclassifications in high-income countries may artificially inflate case numbers. Targeted therapies and immunotherapy have driven progress, but comprehensive epidemiological analyses remain critical for equitable healthcare (206).

CAR-T therapies remain inaccessible in many regions due to cost and infrastructure constraints, a challenge also affecting BsAbs. mRNA-based production could increase access to these therapies (78, 207). However, the global scientific community must still learn how to effectively implement lessons from the COVID-19 pandemic. During that time, the COVID-19 Vaccines Global Access Facility (COVAX) aimed to ensure equitable vaccine distribution but, due to insufficient funding, failed to meet even half of its 2021 target of delivering 2 billion doses, particularly in low-income countries (208).

To date, IVT-mRNA manufacturing has been dominated by three major corporations and their contract manufacturers, primarily based in North America and Europe. In reality, IVT-mRNA technology does not require advanced biologics manufacturing expertise, presenting an opportunity for expansion to new companies and production facilities across Asia, Africa and Latin America (209, 210). While vaccine hesitancy toward IVT-mRNA-based COVID-19 vaccines persists in many low-income countries (as reported by GLP (211)), the technology’s versatility and scalability offer promise for broader applications, including hematological malignancies, potentially enabling more regions to achieve independent production and deployment.

### 5.3 Beyond mRNA: other forms of RNA

Further optimization of mRNA-based therapeutics remains an active area of research, with circular mRNA (circRNA) emerging as a promising platform.

Circular RNA (circRNA), a single-stranded RNA with a covalently closed loop, offers advantages over linear mRNA, including enhanced stability (due to exonuclease resistance), lower immunogenicity, and simplified production. Key elements like internal ribosome entry sites (IRES) and open reading frame (ORF) regions facilitate efficient translation, positioning circRNA as a promising platform for hematologic and other diseases (212–216).

Challenges include declining circularization efficiency with longer sequences and suboptimal methods (e.g., PIE system, T4 RNA ligase), which often yield contaminants. Novel approaches like Clean-PIE and group II intron-based methods are under exploration. Purification remains a hurdle, as current techniques (HPLC, RNase R) are insufficient (217).

Notably, IVT-mRNA optimization strategies do not directly apply to circRNA. For instance, _m1_Ψ modification, beneficial in IVT-mRNA vaccines, offers no advantage for circRNA. Enhancing circRNA translation requires: Locked Nucleic Acids (LNAs) to modulate structure; eIF4G-recruiting aptamers to boost translation initiation, and IRES optimization to improve efficiency, or cap incorporation, as in the work of Wasinska-Kalwa et al. (218).

Proof-of-concept studies demonstrate circRNA-encoded erythropoetin (EPO) sustaining physiological effects in mice for over four days, validating its therapeutic potential (219). However, circRNA’s unique structure demands specialized databases and adapted bioinformatics tools to unlock its full potential (220).

Another innovative direction involves combining IVT-mRNA with regulatory RNA-based strategies, including non-coding RNAs (e.g. siRNA and miRNA) that fine-tune antitumor immunity. For example, synthetic miR-16 mimics (designed to restore the function of this naturally occurring tumor suppressor miRNA) are being evaluated in phase I trials for malignant tumor mesothelioma and non-small lung cancer (NSCLC) (221, 222).

**Figure 5:**
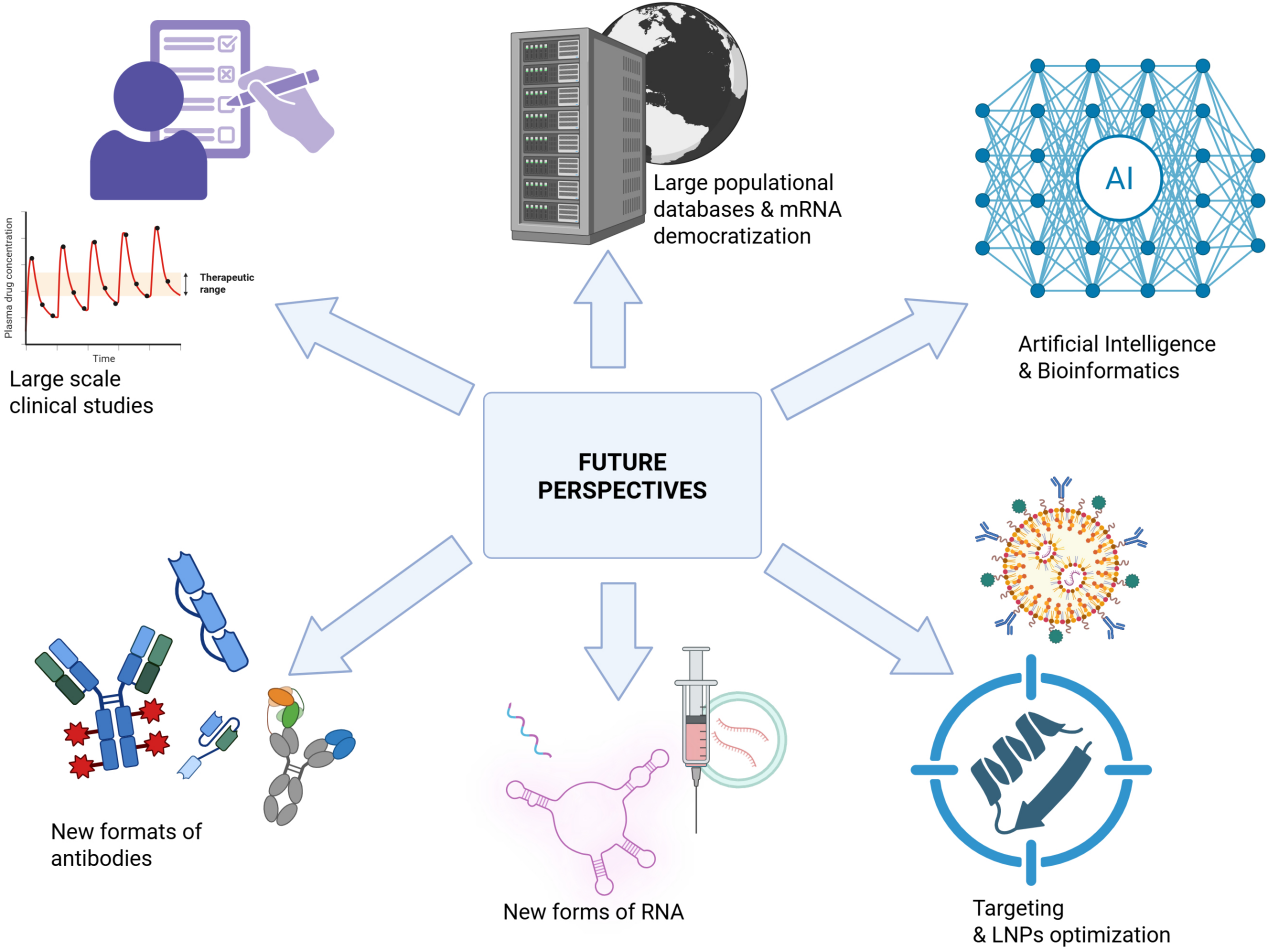
Future perspectives of mRNA technology in hematological malignancies. Created with BioRender.

The future perspectives of this area are summarized in **Figure 5**.

## 6 Conclusions

The vast heterogeneity of hematologic malignancies presents a significant therapeutic challenge, on both clinical and molecular level. The molecular mechanisms underlying these disorders are actively being investigated by research centers worldwide. Immunotherapy has revolutionized hematologic cancer treatment, offering new possibilities for patients. Simultaneously, advancements in therapeutic IVT-mRNA technology have created opportunities for encoding vaccines and anticancer proteins.

The IVT-mRNA technology has been largely accelerated during the COVID-19 pandemic, which drove research centers to optimize production methods. Nonetheless, this monumental leap forward was only possible because it was built upon decades of incremental experimental refinements, like in the work of Krawczyk et al. (223). The same mRNA sequence can behave differently depending on cellular conditions – a challenge highlighted by the work of Kariko and Weissman, who discovered that pseudouridine modification was critical to evading immune detection. This kind of insight would have been nearly impossible to predict computationally without prior empirical evidence.

IVT-mRNA, with its inherent structural and functional advantages, is an ideal platform for delivering vaccines in diseases characterized by high heterogeneity and rapid evolution. Besides infectious diseases, where IVT-mRNA vaccines have become well established, these technologies hold promise for oncology, including hematologic malignancies. However, despite these advantages, an effective cancer vaccine – the *Holy Grail* of oncology – remains undiscovered. Still, never before have researchers been closer to achieving this goal.

Optimizing IVT-mRNA delivery remains a key challenge. LNPs, protein-based carriers, and targeted nanoparticles are among the methods being explored to enhance delivery precision. Continuous improvements aim to balance effective dosing with minimizing the inevitable cytotoxicity.

In summary, IVT-mRNA technology presents a viable alternative to traditional protein-based therapies, including monoclonal antibodies and CAR T cells. Ongoing research will determine whether IVT-mRNA can establish itself as an independent and transformative therapeutic approach in hematologic oncology.

## Footnotes

1
https://www.fda.gov/news-events/press-announcements/fda-approves-and-authorizes-updated-mrna-covid-19-vaccines-better-protect-against-currently, access 05.08.2025.

2
https://www.fda.gov/vaccines-blood-biologics/vaccines/mresvia, access 05.08.2025.

3
https://www.ema.europa.eu/en/medicines/human/EPAR/kostaive, access 05.08.2025.

4 Bhatnagar, V., et al., *FDA Approval Summary: Daratumumab for Treatment of Multiple Myeloma After One Prior Therapy.* Oncologist, 2017. **22**(11): p. 1347-1353.

5 Lonial, S., et al., *Elotuzumab Therapy for Relapsed or Refractory Multiple Myeloma.* N Engl J Med, 2015. **373**(7): p. 621-31.

6 Attal, M., et al., *Isatuximab plus pomalidomide and low-dose dexamethasone versus pomalidomide and low-dose dexamethasone in patients with relapsed and refractory multiple myeloma (ICARIA-MM): a randomised, multicentre, open-label, phase 3 study.* Lancet, 2019. **394**(10214): p. 2096-2107.

7 Kim, Y.H., et al., *Mogamulizumab versus vorinostat in previously treated cutaneous T-cell lymphoma (MAVORIC): an international, open-label, randomised, controlled phase 3 trial.* Lancet Oncol, 2018. **19**(9): p. 1192-1204.

8 Goede, V., et al., *Obinutuzumab plus chlorambucil in patients with CLL and coexisting conditions.* N Engl J Med, 2014. **370**(12): p. 1101-10.

9 Sehn, L.H., et al., *Obinutuzumab plus bendamustine versus bendamustine monotherapy in patients with rituximab-refractory indolent non-Hodgkin lymphoma (GADOLIN): a randomised, controlled, open-label, multicentre, phase 3 trial.* Lancet Oncol, 2016. **17**(8): p. 1081-1093.

10 Wierda, W.G., et al., *Ofatumumab as single-agent CD20 immunotherapy in fludarabine-refractory chronic lymphocytic leukemia.* J Clin Oncol, 2010. **28**(10): p. 1749-55.

11 Hillmen, P., et al., *Chlorambucil plus ofatumumab versus chlorambucil alone in previously untreated patients with chronic lymphocytic leukaemia (COMPLEMENT 1): a randomised, multicentre, open-label phase 3 trial.* Lancet, 2015. **385**(9980): p. 1873-83.

12 McLaughlin, P., et al., *Rituximab chimeric anti-CD20 monoclonal antibody therapy for relapsed indolent lymphoma: half of patients respond to a four-dose treatment program.* J Clin Oncol, 1998. **16**(8): p. 2825-33.

13 Hallek, M., et al., *Addition of rituximab to fludarabine and cyclophosphamide in patients with chronic lymphocytic leukaemia: a randomised, open-label, phase 3 trial.* Lancet, 2010. **376**(9747): p. 1164-74.

14 Salles, G., et al., *Tafasitamab plus lenalidomide in relapsed or refractory diffuse large B-cell lymphoma (L-MIND): a multicentre, prospective, single-arm, phase 2 study.* Lancet Oncol, 2020. **21**(7): p. 978-988.

15 Younes, A., et al., *Results of a pivotal phase II study of brentuximab vedotin for patients with relapsed or refractory Hodgkin's lymphoma.* J Clin Oncol, 2012. **30**(18): p. 2183-9.

16 Pro, B., et al., *Brentuximab vedotin (SGN-35) in patients with relapsed or refractory systemic anaplastic large-cell lymphoma: results of a phase II study.* J Clin Oncol, 2012. **30**(18): p. 2190-6.

17 Bross, P.F., et al., *Approval summary: gemtuzumab ozogamicin in relapsed acute myeloid leukemia.* Clin Cancer Res, 2001. **7**(6): p. 1490-6.

18 Witzig, T.E., et al., *Randomized controlled trial of yttrium-90-labeled ibritumomab tiuxetan radioimmunotherapy versus rituximab immunotherapy for patients with relapsed or refractory low-grade, follicular, or transformed B-cell non-Hodgkin's lymphoma.* J Clin Oncol, 2002. **20**(10): p. 2453-63.

19 Kantarjian, H.M., et al., *Inotuzumab Ozogamicin versus Standard Therapy for Acute Lymphoblastic Leukemia.* N Engl J Med, 2016. **375**(8): p. 740-53.

20 Pennesi, E., et al., *Inotuzumab ozogamicin as single agent in pediatric patients with relapsed and refractory acute lymphoblastic leukemia: results from a phase II trial.* Leukemia, 2022. **36**(6): p. 1516-1524.

21 Caimi, P.F., et al., *Loncastuximab tesirine in relapsed or refractory diffuse large B-cell lymphoma (LOTIS-2): a multicentre, open-label, single-arm, phase 2 trial.* Lancet Oncol, 2021. **22**(6): p. 790-800.

22 Sehn, L.H., et al., *Polatuzumab Vedotin in Relapsed or Refractory Diffuse Large B-Cell Lymphoma.* J Clin Oncol, 2020. **38**(2): p. 155-165.

23 Topp, M.S., et al., *Safety and activity of blinatumomab for adult patients with relapsed or refractory B-precursor acute lymphoblastic leukaemia: a multicentre, single-arm, phase 2 study.* Lancet Oncol, 2015. **16**(1): p. 57-66.

24 Gökbuget, N., et al., *Curative outcomes following blinatumomab in adults with minimal residual disease B-cell precursor acute lymphoblastic leukemia.* Leuk Lymphoma, 2020. **61**(11): p. 2665-2673.

25 Lesokhin, A.M., et al., *Elranatamab in relapsed or refractory multiple myeloma: phase 2 MagnetisMM-3 trial results.* Nat Med, 2023. **29**(9): p. 2259-2267.

26 Thieblemont, C., et al., *Epcoritamab, a Novel, Subcutaneous CD3xCD20 Bispecific T-Cell-Engaging Antibody, in Relapsed or Refractory Large B-Cell Lymphoma: Dose Expansion in a Phase I/II Trial.* J Clin Oncol, 2023. **41**(12): p. 2238-2247.

27 Falchi, L., et al., *Subcutaneous epcoritamab with rituximab+ lenalidomide in patients with relapsed or refractory follicular Lymphoma: Phase 1/2 trial update.* Blood, 2022. **140**(Supplement 1): p. 1464-1466.

28 Dickinson, M.J., et al., *Glofitamab for Relapsed or Refractory Diffuse Large B-Cell Lymphoma.* N Engl J Med, 2022. **387**(24): p. 2220-2231.

29 Budde, L.E., et al., *Safety and efficacy of mosunetuzumab, a bispecific antibody, in patients with relapsed or refractory follicular lymphoma: a single-arm, multicentre, phase 2 study.* Lancet Oncol, 2022. **23**(8): p. 1055-1065.

30 Moreau, P., et al., *Teclistamab in Relapsed or Refractory Multiple Myeloma.* N Engl J Med, 2022. **387**(6): p. 495-505.

31 Chari, A., et al., *Talquetamab, a T-Cell-Redirecting GPRC5D Bispecific Antibody for Multiple Myeloma.* N Engl J Med, 2022. **387**(24): p. 2232-2244.

32 Schinke, C.D., et al., *Pivotal phase 2 MonumenTAL-1 results of talquetamab (tal), a GPRC5DxCD3 bispecific antibody (BsAb), for relapsed/refractory multiple myeloma (RRMM)*. 2023, American Society of Clinical Oncology.

33 Bumma, N., et al., *Linvoseltamab for Treatment of Relapsed/Refractory Multiple Myeloma.* J Clin Oncol, 2024. **42**(22): p. 2702-2712.

34 Neelapu, S.S., et al., *Axicabtagene Ciloleucel CAR T-Cell Therapy in Refractory Large B-Cell Lymphoma.* N Engl J Med, 2017. **377**(26): p. 2531-2544.

35 Jacobson, C.A., et al., *Axicabtagene ciloleucel in relapsed or refractory indolent non-Hodgkin lymphoma (ZUMA-5): a single-arm, multicentre, phase 2 trial.* Lancet Oncol, 2022. **23**(1): p. 91-103.

36 Wang, M., et al., *KTE-X19 CAR T-Cell Therapy in Relapsed or Refractory Mantle-Cell Lymphoma.* N Engl J Med, 2020. **382**(14): p. 1331-1342.

37 Shah, B.D., et al., *KTE-X19 for relapsed or refractory adult B-cell acute lymphoblastic leukaemia: phase 2 results of the single-arm, open-label, multicentre ZUMA-3 study.* Lancet, 2021. **398**(10299): p. 491-502.

38 Berdeja, J.G., et al., *Ciltacabtagene autoleucel, a B-cell maturation antigen-directed chimeric antigen receptor T-cell therapy in patients with relapsed or refractory multiple myeloma (CARTITUDE-1): a phase 1b/2 open-label study.* Lancet, 2021. **398**(10297): p. 314-324.

39 Munshi, N.C., et al., *Idecabtagene Vicleucel in Relapsed and Refractory Multiple Myeloma.* N Engl J Med, 2021. **384**(8): p. 705-716.

40 Abramson, J.S., et al., *Pivotal safety and efficacy results from transcend NHL 001, a multicenter phase 1 study of lisocabtagene maraleucel (liso-cel) in relapsed/refractory (R/R) large B cell lymphomas.* Blood, 2019. **134**: p. 241.

41 Wang, M., et al., *Lisocabtagene Maraleucel in Relapsed/Refractory Mantle Cell Lymphoma: Primary Analysis of the Mantle Cell Lymphoma Cohort From TRANSCEND NHL 001, a Phase I Multicenter Seamless Design Study.* J Clin Oncol, 2024. **42**(10): p. 1146-1157.

42 Siddiqi, T., et al., *Lisocabtagene maraleucel in chronic lymphocytic leukaemia and small lymphocytic lymphoma (TRANSCEND CLL 004): a multicentre, open-label, single-arm, phase 1-2 study.* Lancet, 2023. **402**(10402): p. 641-654.

43 Jabbour, E., et al., *Obecabtagene autoleucel (obe-cel, AUTO1) in adults with relapsed/refractory B-cell acute lymphoblastic leukemia (R/R B-ALL): Overall survival (OS), event-free survival (EFS) and the potential impact of chimeric antigen receptor (CAR)-T cell persistency and consolidative stem cell transplantation (SCT) in the open-label, single-arm FELIX phase Ib/II study*. 2024, American Society of Clinical Oncology.

44 Maude, S.L., et al., *Tisagenlecleucel in Children and Young Adults with B-Cell Lymphoblastic Leukemia.* N Engl J Med, 2018. **378**(5): p. 439-448.

45 Schuster, S.J., et al., *Tisagenlecleucel in Adult Relapsed or Refractory Diffuse Large B-Cell Lymphoma.* N Engl J Med, 2019. **380**(1): p. 45-56.

46 Fowler, N.H., et al., *Tisagenlecleucel in adult relapsed or refractory follicular lymphoma: the phase 2 ELARA trial.* Nat Med, 2022. **28**(2): p. 325-332.

47 Lin L, Cho SF, Xing L, Wen K, Li Y, Yu T, Hsieh PA, Chen H, Kurtoglu M, Zhang Y, Andrew Stewart C, Munshi N, Anderson KC, Tai YT. Preclinical evaluation of CD8+ anti-BCMA mRNA CAR T cells for treatment of multiple myeloma. Leukemia. 2021 Mar;35(3):752-763. doi: 10.1038/s41375-020-0951-5. Epub 2020 Jul 6. PMID: 32632095; PMCID: PMC7785573.

48 Svoboda J, Rheingold SR, Gill SI, Grupp SA, Lacey SF, Kulikovskaya I, Suhoski MM, Melenhorst JJ, Loudon B, Mato AR, Nasta SD, Landsburg DJ, Youngman MR, Levine BL, Porter DL, June CH, Schuster SJ. Nonviral RNA chimeric antigen receptor-modified T cells in patients with Hodgkin lymphoma. Blood. 2018 Sep 6;132(10):1022-1026. doi: 10.1182/blood-2018-03-837609. Epub 2018 Jun 20. PMID: 29925499.

49
https://clinicaltrials.gov/study/NCT02623582, access 21.09.2025

50 Timothy A. Yap et al. First-in-human phase I/II trial evaluating BNT142, a first-in-class mRNA encoded, bispecific antibody targeting Claudin 6 (CLDN6) and CD3, in patients (pts) with CLDN6-positive advanced solid tumors. JCO 43, 2501-2501(2025). DOI:10.1200/JCO.2025.43.16_suppl.2501

51 Neelapu SS, Jacobson CA, Ghobadi A, Miklos DB, Lekakis LJ, Oluwole OO, Lin Y, Braunschweig I, Hill BT, Timmerman JM, Deol A, Reagan PM, Stiff P, Flinn IW, Farooq U, Goy AH, McSweeney PA, Munoz J, Siddiqi T, Chavez JC, Herrera AF, Bartlett NL, Bot AA, Shen RR, Dong J, Singh K, Miao H, Kim JJ, Zheng Y, Locke FL. Five-year follow-up of ZUMA-1 supports the curative potential of axicabtagene ciloleucel in refractory large B-cell lymphoma. Blood. 2023 May 11;141(19):2307-2315. doi: 10.1182/blood.2022018893. PMID: 36821768; PMCID: PMC10646788.

52 Schuster SJ, Bishop MR, Tam CS, Waller EK, Borchmann P, McGuirk JP, Jäger U, Jaglowski S, Andreadis C, Westin JR, Fleury I, Bachanova V, Foley SR, Ho PJ, Mielke S, Magenau JM, Holte H, Pantano S, Pacaud LB, Awasthi R, Chu J, Anak Ö, Salles G, Maziarz RT; JULIET Investigators. Tisagenlecleucel in Adult Relapsed or Refractory Diffuse Large B-Cell Lymphoma. N Engl J Med. 2019 Jan 3;380(1):45-56. doi: 10.1056/NEJMoa1804980. Epub 2018 Dec 1. PMID: 30501490.

53 Litzow MR, Sun Z, Mattison RJ, Paietta EM, Roberts KG, Zhang Y, Racevskis J, Lazarus HM, Rowe JM, Arber DA, Wieduwilt MJ, Liedtke M, Bergeron J, Wood BL, Zhao Y, Wu G, Chang TC, Zhang W, Pratz KW, Dinner SN, Frey N, Gore SD, Bhatnagar B, Atallah EL, Uy GL, Jeyakumar D, Lin TL, Willman CL, DeAngelo DJ, Patel SB, Elliott MA, Advani AS, Tzachanis D, Vachhani P, Bhave RR, Sharon E, Little RF, Erba HP, Stone RM, Luger SM, Mullighan CG, Tallman MS. Blinatumomab for MRD-Negative Acute Lymphoblastic Leukemia in Adults. N Engl J Med. 2024 Jul 25;391(4):320-333. doi: 10.1056/NEJMoa2312948. PMID: 39047240; PMCID: PMC11334054.

54 Teclistamab in Relapsed or Refractory Multiple Myeloma, P. Moreau, L. Garfall Alfred, W. C. J. van de Donk Niels, H. Nahi, F. San-Miguel Jesús, A. Oriol, et al. New England Journal of Medicine 2022 Vol. 387 Issue 6 Pages 495-505, DOI: 10.1056/NEJMoa2203478 https://doi.org/10.1056/NEJMoa2203478

55 Chari A, Touzeau C, Schinke C, Minnema MC, Berdeja JG, Oriol A, van de Donk NWCJ, Rodríguez-Otero P, Morillo D, Martinez-Chamorro C, Mateos MV, Costa LJ, Caers J, Rasche L, Krishnan A, Ye JC, Karlin L, Lipe B, Vishwamitra D, Skerget S, Verona R, Ma X, Qin X, Ludlage H, Campagna M, Masterson T, Hilder B, Tolbert J, Renaud T, Goldberg JD, Kane C, Heuck C, San-Miguel J, Moreau P. Safety and activity of talquetamab in patients with relapsed or refractory multiple myeloma (MonumenTAL-1): a multicentre, open-label, phase 1-2 study. Lancet Haematol. 2025 Apr;12(4):e269-e281. doi: 10.1016/S2352-3026(24)00385-5. Epub 2025 Mar 13. PMID: 40090350.

56 Linton KM, Vitolo U, Jurczak W, Lugtenburg PJ, Gyan E, Sureda A, Christensen JH, Hess B, Tilly H, Cordoba R, Lewis DJ, Okada C, Hutchings M, Clausen MR, Sancho JM, Cochrane T, Leppä S, Chamuleau MED, Gernhardt D, Altıntaş I, Liu Y, Ahmadi T, Dinh MH, Hoehn D, Favaro E, Elliott B, Thieblemont C, Vose JM. Epcoritamab monotherapy in patients with relapsed or refractory follicular lymphoma (EPCORE NHL-1): a phase 2 cohort of a single-arm, multicentre study. Lancet Haematol. 2024 Aug;11(8):e593-e605. doi: 10.1016/S2352-3026(24)00166-2. Epub 2024 Jun 15. PMID: 38889737.

57 Phillips TJ, Carlo-Stella C, Morschhauser F, Bachy E, Crump M, Trněný M, Bartlett NL, Zaucha J, Wrobel T, Offner F, Humphrey K, Relf J, Filézac de L'Etang A, Carlile DJ, Byrne B, Qayum N, Lundberg L, Dickinson M. Glofitamab in Relapsed/Refractory Mantle Cell Lymphoma: Results From a Phase I/II Study. J Clin Oncol. 2025 Jan 20;43(3):318-328. doi: 10.1200/JCO.23.02470. Epub 2024 Oct 4. PMID: 39365960; PMCID: PMC11771347.

58 Brenner, S., F. Jacob, and M. Meselson, *An unstable intermediate carrying information from genes to ribosomes for protein synthesis.* Nature, 1961. 190: p. 576-581.

59 Gurdon, J.B., et al., *Use of frog eggs and oocytes for the study of messenger RNA and its translation in living cells.* Nature, 1971. 233(5316): p. 177-82.

60 Darnell, J.E., R. Wall, and R.J. Tushinski, *An adenylic acid-rich sequence in messenger RNA of HeLa cells and its possible relationship to reiterated sites in DNA.* Proc Natl Acad Sci U S A, 1971. 68(6): p. 1321-5.

61 Moyer, S.A., et al., *Methylated and blocked 5' termini in vesicular stomatitis virus in vivo mRNAs.* Cell, 1975. 5(1): p. 59-67.

62 Melton, D.A., et al., *Efficient in vitro synthesis of biologically active RNA and RNA hybridization probes from plasmids containing a bacteriophage SP6 promoter.* Nucleic Acids Res, 1984. 12(18): p. 7035-56.

63 Malone, R.W., P.L. Felgner, and I.M. Verma, *Cationic liposome-mediated RNA transfection.* Proc Natl Acad Sci U S A, 1989. 86(16): p. 6077-81.

64 Wolff, J.A., et al., *Direct gene transfer into mouse muscle in vivo.* Science, 1990. 247(4949 Pt 1): p. 1465-8.

65 Martinon, F., et al., *Induction of virus-specific cytotoxic T lymphocytes in vivo by liposome-entrapped mRNA.* Eur J Immunol, 1993. 23(7): p. 1719-22.

66 Heiser, A., et al., *Autologous dendritic cells transfected with prostate-specific antigen RNA stimulate CTL responses against metastatic prostate tumors.* J Clin Invest, 2002. 109(3): p. 409-17.

67 Yoon, S.H., et al., *Adoptive immunotherapy using human peripheral blood lymphocytes transferred with RNA encoding Her-2/neu-specific chimeric immune receptor in ovarian cancer xenograft model.* Cancer Gene Ther, 2009. 16(6): p. 489-97.

68 Beatty, G.L., et al., *Mesothelin-specific chimeric antigen receptor mRNA-engineered T cells induce anti-tumor activity in solid malignancies.* Cancer Immunol Res, 2014. 2(2): p. 112-20.

69 Stadler, C.R., et al., *Elimination of large tumors in mice by mRNA-encoded bispecific antibodies.* Nat Med, 2017. 23(7): p. 815-817.

70 Billingsley, M.M., et al., *Ionizable Lipid Nanoparticle-Mediated mRNA Delivery for Human CAR T Cell Engineering.* Nano Lett, 2020. 20(3): p. 1578-1589.

71 Huang, C., et al., *Lipid Nanoparticle Delivery System for mRNA Encoding B7H3-redirected Bispecific Antibody Displays Potent Antitumor Effects on Malignant Tumors.* Adv Sci (Weinh), 2023. 10(3): p. e2205532.

72 Hu, Q., et al., *Scarless circular mRNA-based CAR-T cell therapy elicits superior anti-tumor efficacy.* bioRxiv, 2024: p. 2024.08.05.606578.

73 Huang, Y., et al., *1342 Organ-specific delivery of a mRNA-encoded bispecific T cell engager targeting Glypican-3 in hepatocellular carcinoma*. 2024, BMJ Specialist Journals.
